# Clostridium ramosum regulates enterochromaffin cell development and serotonin release

**DOI:** 10.1038/s41598-018-38018-z

**Published:** 2019-02-04

**Authors:** Ana D. Mandić, Anni Woting, Tina Jaenicke, Anika Sander, Wiebke Sabrowski, Ulrike Rolle-Kampcyk, Martin von Bergen, Michael Blaut

**Affiliations:** 1 Department of Gastrointestinal Microbiology, German Institute of Human Nutrition Potsdam-Rehbruecke, Nuthetal, Germany; 2 Department of Molecular Systems Biology, Helmholtz Centre for Enviromental Research –UFZ, Leipzig, Germany; 3 Institute of Biochemistry, Faculty of Life Sciences, University of Leipzig, Leipzig, Germany

## Abstract

Peripheral serotonin (5-hydroxytryptamine: 5-HT) synthesized in the intestine by enterochromaffin cells (ECs), plays an important role in the regulation of peristaltic of the gut, epithelial secretion and promotes the development and maintenance of the enteric neurons. Recent studies showed that the indigenous gut microbiota modulates 5-HT signalling and that ECs use sensory receptors to detect dietary and microbiota-derived signals from the lumen to subsequently transduce the information to the nervous system. We hypothesized that *Clostridium ramosum* by increasing gut 5-HT availability consequently contributes to high-fat diet-induced obesity. Using germ-free mice and mice monoassociated with *C*. *ramosum*, intestinal cell lines and mouse organoids, we demonstrated that bacterial cell components stimulate host 5-HT secretion and program the differentiation of colonic intestinal stem progenitors toward the secretory 5-HT-producing lineage. An elevated 5-HT level regulates the expression of major proteins involved in intestinal fatty acid absorption *in vitro*, suggesting that the presence of *C*. *ramosum* in the gut promotes 5-HT secretion and thereby could facilitates intestinal lipid absorption and the development of obesity.

## Introduction

*Clostridium ramosum* is an anaerobic, spore-forming, Gram-positive bacterium that has been linked to obesity in humans^1–3^. We have previously shown that mice associated with a simplified human intestinal microbiota composed of eight bacterial species including *C*. *ramosum* (SIHUMI) as well as mice monoassociated with *C*. *ramosum* (Cra) are more prone to obesity development on a high-fat diet (HFD) compared to mice lacking *C*. *ramosum* (SIHUMIw/oCra)^4^. When fed a low-fat diet (LFD) mice stayed lean independently of their microbial status. After four weeks of HFD feeding SIHUMI and Cra mice gained significantly more body weight, body fat and higher liver triglyceride concentrations than HFD-fed SIHUMIw/oCra mice^4^. Given that the mechanism underlying the obesogenic effect of *C*. *ramosum* is still obscure, we investigated potential mechanistic links by comparing germ-free (GF) and Cra mice fed either HFD or LFD.

Serotonin (5-hydroxytryptamine [5-HT]) is a monoaminergic neurotransmitter that constitutes an important signaling molecule in both brain and periphery. More than 90% of 5-HT in the body is synthesized in the gut by specific enteroendocrine cells referred to as enterochromaffin cells (ECs). Following its formation from tryptophan by the rate-limiting enzyme tryptophan hydroxylase 1 (TPH1) and the ensuing 5-hydroxytryptophan decarboxylase, 5-HT is packed into vesicles by the vesicular monoamine transporter. 5-HT is released from the vesicles either near the apical membrane into the gut lumen or near the basal border into the lamina propria, where it interacts with nerve terminals and immune cells to finally being taken up by the platelets^5^. Clearance of 5-HT is furthermore mediated by its transport into epithelial cells by serotonin re-uptake transporters (SERT), which are present in both apical and basal membranes. Once taken up 5-HT is metabolized by monoamine oxidase (MAO) and aldehyde dehydrogenase resulting in various products, with 5-hydroxyindole acetic acid being the most abundant one^6^. Only 2% of 5-HT in blood is present in its free form and partially originates from pancreatic β cells, adipocytes and osteoclasts^7^.

Peripheral 5-HT affects gastrointestinal motility and secretion of digestive enzymes, facilitates wound healing^8^, visceral hypersensitivity^9^, recruits neutrophils to the site of acute inflammation, stimulates production of pro-inflammatory cytokines^10^ and inhibits bone formation^11^. Interestingly, with respect to obesity 5-HT has opposite effects in brain and peripheral organs. Brain-produced 5-HT has been considered as a target against obesity since it has a strong anorectic effect^12–14^, whereas increased levels of peripheral 5-HT are associated with the weight gain and adiposity in mice and rats^15–17^. Several genome-wide association studies in humans have linked the serotonergic system to obesity^14^. Single nucleotide polymorphisms in *Tph1* and the genes of 5-HT receptors were significantly associated with obesity^18–20^. Furthermore, recent human study showed that obese humans have increased capacity to produce and release 5-HT in the small intestine^21^. On the one hand fat-rich diets were reported to increase 5-HT production in rats fed a Western diet and in mice fed a HFD^15,17^ and on the other hand increased levels of 5-HT in plasma were also observed during fasting with values being much higher than usually observed^22,23^. Accumulating evidence indicates that the gut microbiota plays an important role in controlling 5-HT availability through the effects of short-chain fatty acids^24^ secondary bile acids and several microbiota-derived metabolites^25^. By signaling to colonic enterochromaffin cells, these molecules probably promote 5-HT biosynthesis^25^. Since obesity is linked to shifts in intestinal microbial community composition in both humans and mice^26–28^, the gut microbiota could be a missing link to understand the interdependence between nutrition, 5-HT signaling and its effects on metabolic diseases such as obesity.

In the light of these recent findings we hypothesized that *C*. *ramosum* promotes obesity by modulating 5-HT availability in the intestinal epithelium. In this study, we used mice that were germ-free or monoassociated with *C*. *ramosum*, intestinal and pancreatic cell lines, as well as small intestinal and colonic mouse organoids to demonstrate that the cell components of *C*. *ramosum* stimulate 5-HT secretion from enterochromaffin cells by promoting differentiation of intestinal stem progenitors toward the secretory 5-HT-producing lineage. Since elevated 5-HT levels enhance the expression of several proteins involved in intestinal fatty acid absorption *in vitro* and we observed increased expression of those proteins in HFD-fed mice monoassociated with *C*. *ramosum*, we proposed that the presence of *C*. *ramosum* increases intestinal 5-HT production and thereby could favor the absorption of fatty acids and the development of obesity.

## Results

### *Clostridium ramosum* has a mild effect on obesity development in gnotobiotic mice fed a semisynthetic HFD

**Figure 1:**
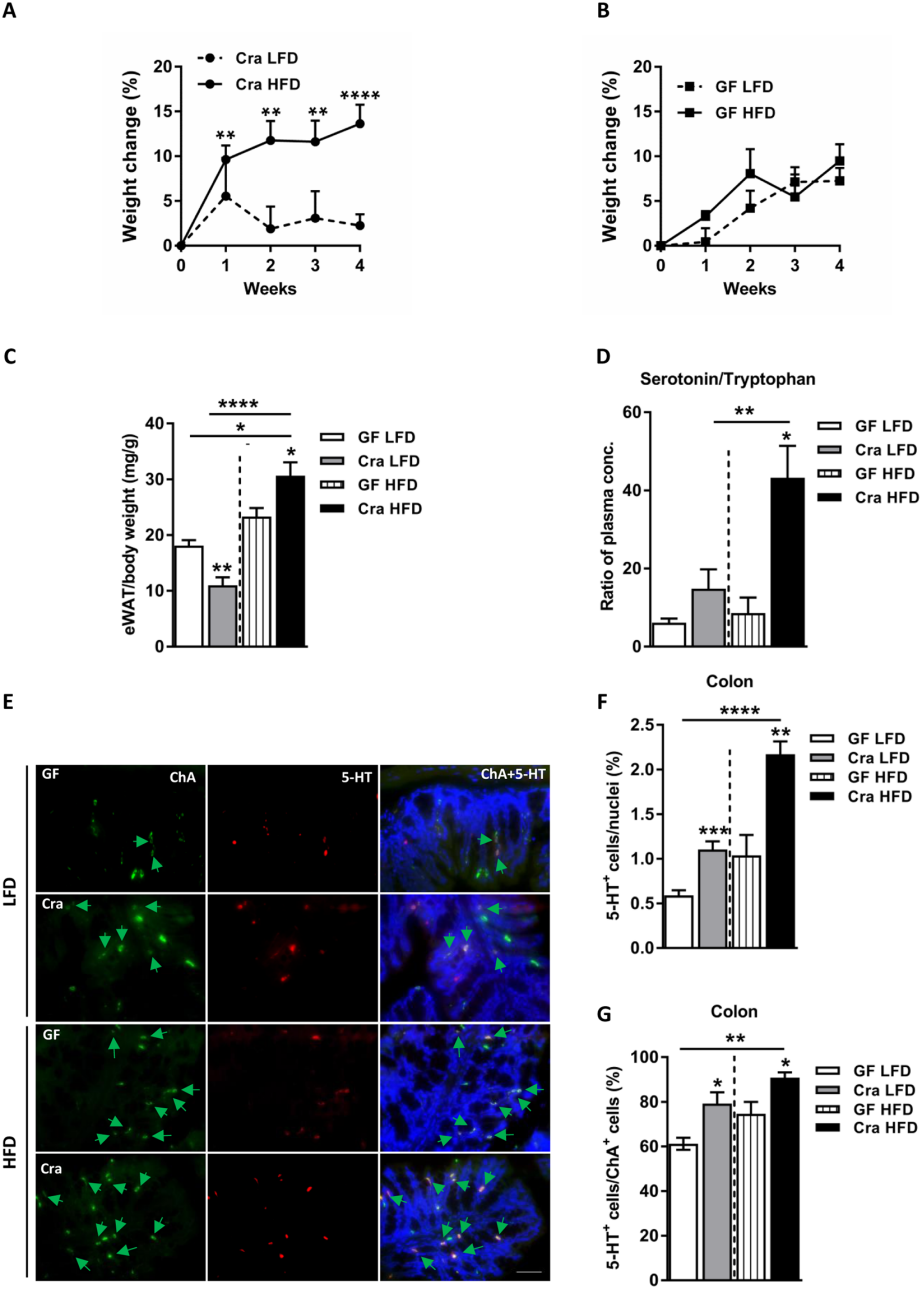
*Clostridium ramosum* promotes obesity in mice monoassociated with this bacterium (Cra) compared to germ-free (GF) mice after 4 weeks of high-fat diet feeding. (**A**,**B**) Weight change after 4 weeks of either LFD or HFD feeding. (**C**) Relative weight of eWAT after 4 weeks of LFD or HFD feeding. (**D**) Ratio of plasma serotonin to tryptophan concentrations from Cra and GF mice fed either LFD or HFD. (**E**) Representative colonic immunofluorescence images stained for serotonin (5-HT), chromogranin A (ChA) and merged. Arrows indicate double-positive cells stained for 5-HT and ChA. Scale bar = 50 µm. (**F**) Quantification of colonic 5-HT-positive (5-HT^+^) cells presented as percentage of total cell number (nuclei) of indicated mouse groups. (**G**) Percentage of double-positive (5-HT^+^/ChA^+^) cells. Means ± SEM of n = 5–12 per group are shown. Asterisks above bars indicate statistical differences between Cra and GF mice for each diet group analyzed by Mann-Whitney U test. Differences among all 4 groups analyzed by Kruskal-Wallis and Dunn’s Post-hoc tests and are indicated by lines. *p < 0.05; **p < 0.01; ***p < 0.001; ****p < 0.0001. See also Fig. S1.

Mice monoassociated with *Clostridium ramosum* (Cra) displayed a higher relative change in body weight after 4 weeks of HFD feeding compared to LFD-fed Cra mice (13.64 ± 2.12% versus 2.25 ± 1.26% respectively, p < 0.0001) (Figs 1A and S1J), increased total body fat percentages (36.13 ± 0.56% versus 27.80 ± 0.64% respectively, p < 0.05) (Fig. S1A) and increased epididymal white adipose tissue weights (eWAT) (for comparison between HFD-fed Cra and LFD-fed Cra mice: 30.63 ± 2.42 versus 10.98 ± 1.44 mg/g body weight, respectively, p < 0.0001; for comparison between HFD-fed Cra and HFD-fed GF mice: 30.63 ± 2.42 versus 23.33 ± 1.52 mg/g body weight, respectively, p < 0.05) (Fig. 1C), whereas germ-free (GF) mice had similar relative body-weight changes in both diet groups during intervention (Fig. 1B) with no differences in total body fat or eWAT weight (Figs 1C and S1A). Furthermore, HFD-fed Cra mice also had increased weight of subcutaneous white adipose tissue (sWAT) compared to LFD-fed Cra mice (13.93 ± 0.89 versus 7.37 ± 0.72 mg/g body weight, respectively, p < 0.001; Fig. S1B), increased mesenteric white adipose tissue (mWAT) weight (16.33 ± 0.88 versus 10.38 ± 0.72 mg/g body weight, respectively, p < 0.001) (Fig. S1C) and higher blood glucose levels (Fig. S1E). Surprisingly, LFD-fed Cra mice had lower brown adipose tissue weights, total body fat, eWAT, sWAT, mWAT and blood glucose levels compared to GF mice fed the same diet (Figs 1C and S1A–E) but higher total body lean masses (Fig. S1I). HFD-fed Cra mice displayed reduced small intestinal length compared to HFD-fed GF mice (Fig. S1G) and lower full cecum weight compared to GF mice independent of the diet (Fig. S1H). No differences between the groups were observed in liver weight or liver metabolism (data not shown). Importantly, the mouse groups did not differ in their energy intakes (data not shown).

### *Clostridium ramosum* increases host 5-HT availability

**Figure 2:**
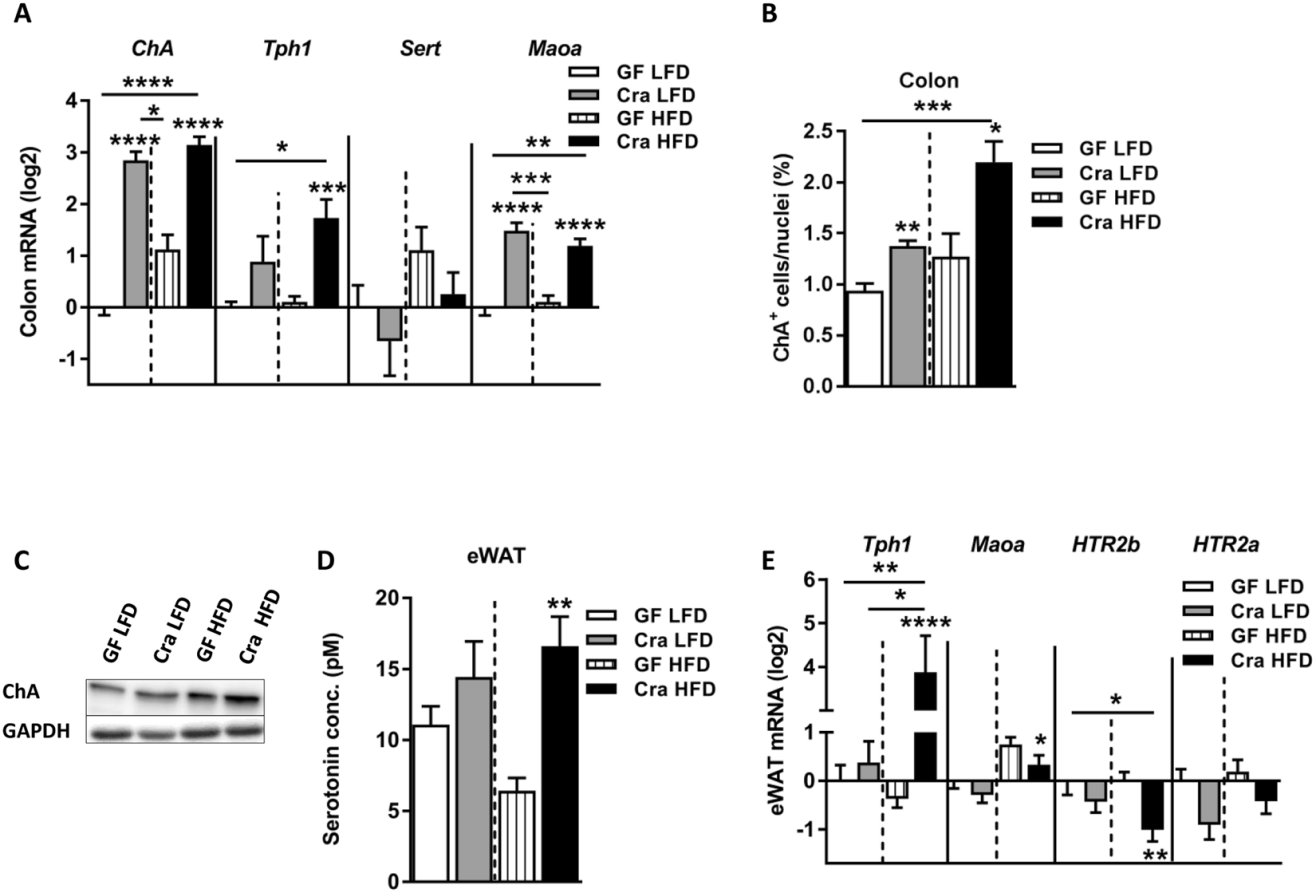
*Clostridium ramosum* regulates peripheral 5-HT availability. (**A**) mRNA expression in colonic tissue derived from Cra and GF mice after 4 weeks of feeding LFD or HFD determined for chromogranin A (*ChA*), tryptophan hydroxylase 1 (*Tph1*), serotonin transporter (*Sert*) and monoamine oxidase A (*Maoa*). (**B**) Quantification of colonic ChA-positive (ChA^+^) enteroendocrine cells presented as percentage of total cell number (nuclei) of indicated mouse groups. Means ± SEM of n = 8–13 per group are shown. *p < 0.05. (**C**) Western blot protein analysis with a ChA-specific antibody (86 kDa) applied to colonic mucosa isolated from indicated mice. GAPDH (36 kDa) was used as a loading control. (**D**) 5-HT concentration in eWAT cell lysates from Cra and GF mice fed either LFD or HFD for 4 weeks. Means ± SEM of n = 5–8 per group are shown. **p < 0.01. (**E**) mRNA expression in eWAT from Cra and GF mice after 4 weeks of feeding either LFD or HFD for *Tph1*, *Maoa*, 5-hydroxytryptamine receptor 2B (*HTR2b*) and 5-hydroxytryptamine receptor 2A (*HTR2a*). (**A**,**E**) Bars indicate mean ± SEM log2 fold change compared to mean of LFD-fed GF group. Asterisks above bars indicate statistical differences between Cra and GF mice for an each diet group analyzed by Mann-Whitney U test. Differences among all 4 groups were analyzed by Kruskal-Wallis and Dunn’s Post-hoc tests and are indicated by lines. n = 8–13. *p < 0.05; **p < 0.01; ***p < 0.001; ****p < 0.0001. See also Fig. S2.

After four weeks of dietary intervention the plasma serotonin (5-HT)/tryptophan ratio in HFD-fed Cra mice was higher compared to HFD-fed GF mice or LFD-fed Cra mice (p < 0.05; p < 0.01 respectively) (Fig. 1D). Moreover, the levels of 5-HT in plasma of HFD-fed Cra mice were higher than those in LFD-fed Cra mice (p < 0.05), although just mildly elevated when compared to GF HFD mice (p = 0.3834) (Fig. S2A). While plasma tryptophan levels did not differ significantly between the groups (Fig. S2B), the kynurenine/tryptophan ratio was lower in Cra mice, independently of the diet (p < 0.05; p < 0.001 respectively) (Fig. S2C). In agreement with these results immunofluorescence staining revealed higher numbers of colonic serotonin-producing (5-HT^+^) and chromogranin A-positive (ChA^+^) enteroendocrine cells in Cra mice compared to GF mice, both fed LFD (for single positive 5-HT^+^ cells: p < 0.001, for double positive cells: p < 0.05) and HFD (for single positive 5-HT^+^ cells: p < 0.01, for double positive cells: p < 0.01) (Fig. 1E–G). Furthermore, Cra mice showed higher numbers of colonic ChA^+^ cells compared to GF mice based on gene-expression analysis (p < 0.0001) (Fig. 2A), immunofluorescence staining (for LFD: p < 0.01, for HFD: p < 0.05) (Fig. 2B) and protein analysis (Figs 2C and S3E), indicating that Cra mice have an increased number of enteroendocrine cells. Since the majority of ChA^+^ cells stained also positive for 5-HT, we investigated whether *C*. *ramosum* affects 5-HT availability in the intestine. Genes involved in 5-HT metabolism were analyzed and we observed an increased gene expression of ileal and colonic tryptophan hydroxylase 1(*Tph1*) and monoamine oxidase A (*Maoa*) in Cra mice compared to GF mice, both fed HFD, both indicating an increased 5-HT production and clearance in Cra mice. Gene expression of the 5-HT transporter *Sert* was slightly reduced in colon and even significantly reduced in ileum of HFD-fed Cra mice compared to HFD-fed GF mice (Figs 2A and S2E). The latter probably constitutes a counter regulation preventing extensive 5-HT accumulation as described previously^25^.

Elevated 5-HT levels were also detected in eWAT of HFD-fed Cra mice compared to HFD-fed GF mice (p < 0.01) (Fig. 2D) which is in accordance with increased *Tph1* and reduced *Htr2b* (5-HT receptor) mRNA levels in eWAT (Fig. 2E). The modest reduction in *Maoa* gene expression observed in eWAT of HFD-fed Cra mice (Fig. 2E) differs from the results obtained for intestinal tissue and so far is not explainable.

### *Clostridium ramosum* increases 5-HT availability in intestinal cell lines and organoids

**Figure 3:**
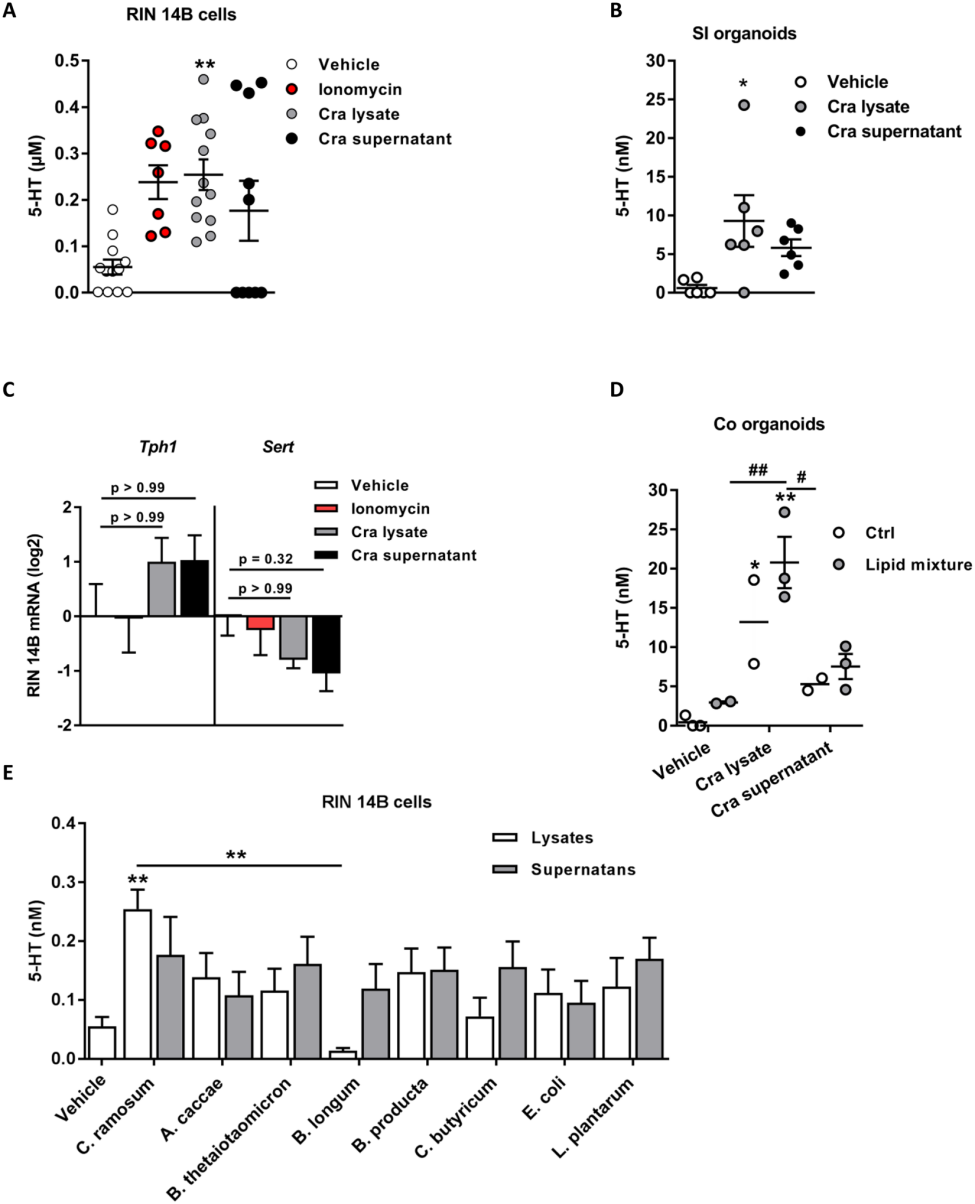
*In vitro* 5-HT release is stimulated by *Clostridium ramosum*. (**A**) Level of 5-HT released from RIN14B cells after exposure to *C*. *ramosum* supernatant, heat inactivated cell lysate or to ionomycin for 1 h. Means ± SEM of n = 7–12 per group are shown. (**B**) Level of 5-HT released from mouse small intestinal organoids after exposure to culture supernatant or heat inactivated cell lysate of *C*. *ramosum* for 7 days. Means ± SEM of n = 6 per group are shown. (**C**) mRNA expression of *Tph1* and *SERT* derived from RIN14B cells 1 h after exposure to culture supernatant or heat killed lysate of *C*. *ramosum*, or to ionomycin. (**D**) Level of 5-HT released from mouse colonic organoids after exposure to culture supernatant or heat inactivated cell lysate of *C*. *ramosum* and 2% lipid mixture. Means ± SEM of n = 2–3 per group are shown. Differences among all 6 groups were analyzed by one-way Anova and Tukey’s multiple comparison tests. *p < 0.05; **p < 0.01. ^#^p < 0.05; ^##^p < 0.01 for Cra lysates + lipid mixture versus vehicle + lipid mixture and Cra supernatant respectively. (**E**) Level of 5-HT released from RIN14B cells after exposure to supernatants or heat inactivated cell lysates of *Anaerostipes caccae*, *Bacteroides thetaiotaomicron*, *Bifidobacterium longum*, *Blautia product*, *Clostridium butyricum*, *Escherichia* coli, *Clostridium ramosum* and *Lactobacillus plantarum* and vehicle (BHI:YCFA medium). Means ± SEM of n = 6 per group are shown. Differences among all groups were analyzed by Kruskal-Wallis and Dunn’s Post-hoc tests. *p < 0.05; **p < 0.01.

To investigate the direct effects of *C*. *ramosum* on 5-HT production, RIN14B chromaffin cells and mouse small intestinal (SI) and colonic organoids were incubated for 1 h with supernatants from bacterial suspensions containing *C*. *ramosum* grown to the late exponential phase, cell lysates or the calcium ionophore ionomycin as a positive control, triggering 5-HT exocytosis through voltage-gated Ca^2+^ channels. Stimulation of chromaffin cells and organoids with lysates of *C*. *ramosum* cells led to an increased release of 5-HT (for ECs: lysates versus vehicle: 0.25 ± 0.03 µM versus 0.05 ± 0.01 µM, p < 0.01; for SI organoids: lysates versus vehicle: 9.28 ± 3.33 nM versus 0.60 ± 0.39 nM, p < 0.05; for colonic organoids: lysates versus vehicle: 13.22 ± 5.34 nM versus 0.45 ± 0.46 nM, p < 0.05) (Fig. 3A,B and D). Although 5-HT levels were elevated after stimulation with bacterial supernatants the observed differences did not reach statistical significance (for ECs: supernatants versus vehicle: 0.17 ± 0.06 µM versus 0.05 ± 0.01 µM, for SI organoids: supernatants versus vehicle: 5.81 ± 1.07 nM versus 0.60 ± 0.39 nM; for colonic organoids: 5.28 ± 0.79 nM versus 0.45 ± 0.46 nM). Genes involved in 5-HT metabolism were not affected (Fig. 3C), suggesting that *C*. *ramosum* or some of its cell components drive 5-HT secretion by a yet unknown mechanism rather than stimulate de novo 5-HT synthesis directly.

To examine whether HFD in combination with intestinal microbiota enhances 5-HT secretion we stimulated colonic organoids with a 2% lipid mixture together with Cra lysates or supernatants, as shown in Fig. 3D, treatment with lipids slightly increased 5-HT release.

Given that SIHUMI mice are more prone to obesity compared to mice lacking *C*. *ramosum* (SIHUMIw/oCra)^4^, we stimulated RIN14B cells for 1 h with 8 supernatants and lysates from bacterial suspensions containing each member of SIHUMI consortium grown to the late exponential phase to determine whether other members of SIHUMI consortium have similar effects on host 5-HT availability. As shown in Fig. 3D only *C*. *ramosum* lysates were able to stimulate 5-HT secretion compared to control (p < 0.01).

**Figure 4:**
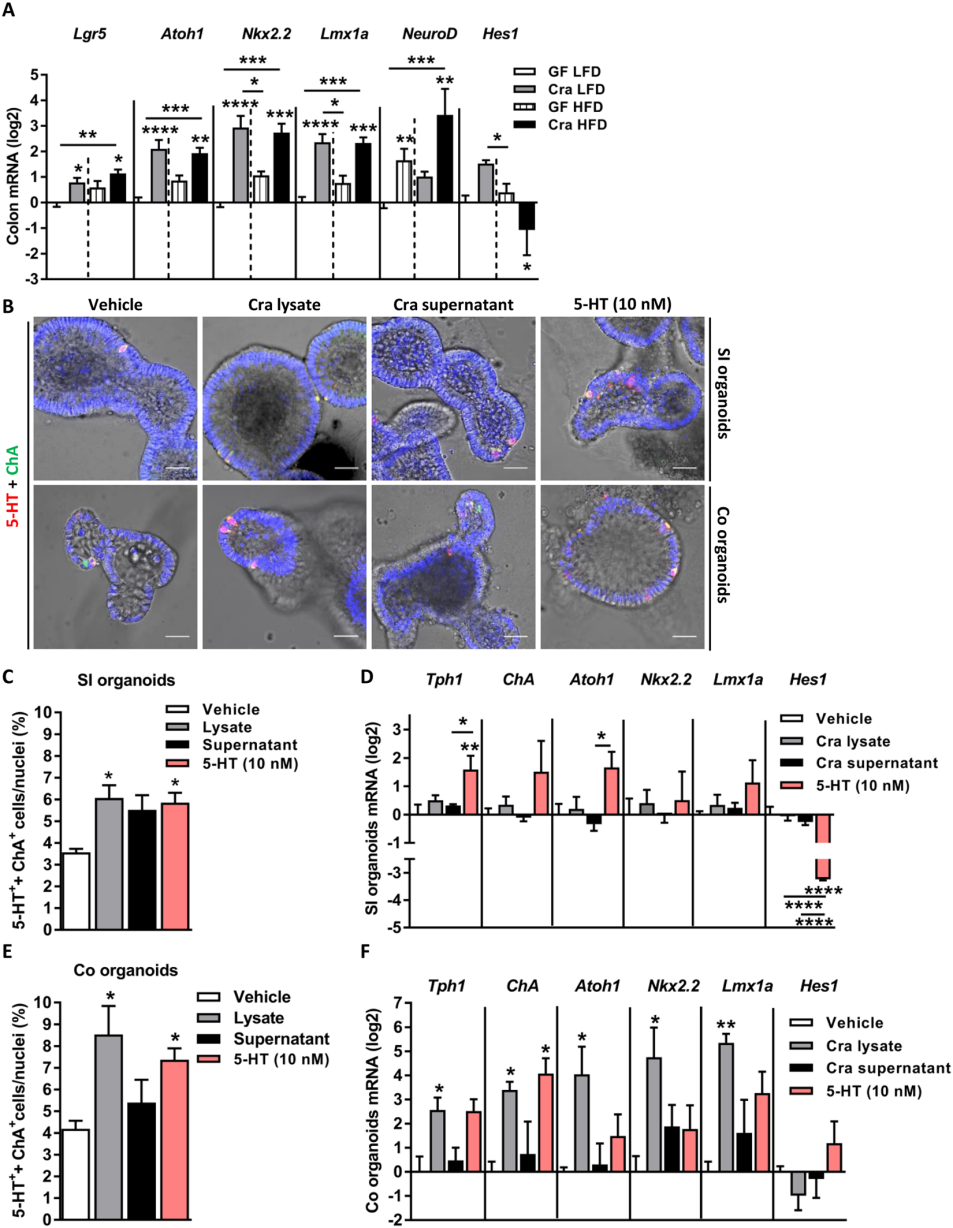
*Clostridium ramosum* induces expansion of enterochromaffin cells. (**A**) Expression of genes encoding stem-cell marker leucine-rich repeat-containing G-protein coupled receptor 5 (*Lgr5*) and transcription factors involved in enterochromaffin cell development: protein atonal homolog 1 (*Atoh1*), homeobox protein Nkx2.2 (*Nkx2*.*2*), LIM homeobox transcription factor 1 alpha (*Lmx1a*), neuronal differentiation (*NeuroD*) and hairy and enhancer of split-1 (*Hes1*) in colon of Cra and GF mice fed either LFD or HFD. Bars indicate mean ± SEM log2 fold change compared to mean of LFD-fed GF group. Asterisks above bars indicate statistical differences between Cra and GF mice for each diet group analyzed by Mann-Whitney U test. Differences among all 4 groups were analyzed by Kruskal-Wallis and Dunn’s Post-hoc tests and are indicated by lines. n = 8–13. *p < 0.05; **p < 0.01; ***p < 0.001; ****p < 0.0001. (**B**) Representative immunofluorescence images stained for 5-HT, ChA and 4′,6-diamidino-2-phenylindole (DAPI) in small intestinal and colonic organoids after exposure to culture supernatant or heat inactivated cell lysate *C*. *ramosum*, or 5-HT (10 nM) for 7 days. Scale bar = 30 µm. (**C**,**E**) Quantification of double positive (5-HT^+^/ChA^+^) enterochromaffin cells from small intestinal and colonic organoids. Means ± SEM of n = 4–6 per group are shown. (**D**,**F**) mRNA levels of *Tph1*, *ChA*, *Atoh1*, *Nkx2*.*2*, *Lmx1a* and *Hes1* in small intestinal and colonic organoids. Bars indicate mean ± SEM log2 fold change compared to mean of vehicle-treated controls (n = 3–4). Differences among all 4 groups were analyzed by one-way Anova and Tukey’s multiple comparison tests and are indicated by lines. *p < 0.05; **p < 0.01; ***p < 0.001; ****p < 0.0001. See also Fig. S3.

It has recently been demonstrated that interleukin 33 (IL-33) takes advantage of intestinal stem cell pluripotency to program intestinal progenitor cells to differentiate toward the secretory lineage in response to *Salmonella enterica* ser. Typhimurium infection resulting in an expansion of Paneth and goblet cells^29^. *Helicobacter pylori* colonizes stem and progenitor cell compartments leading to increased stemness and glandular hyperplasia^30^. To determine whether the effects of *C*. *ramosum* on host 5-HT availability depend on a similar mechanism, mRNA expression of a stem-cell marker and of several central transcription factors involved in the development of intestinal cells was analyzed by qPCR. Colonic mRNAs for leucine-rich repeat-containing G-protein-coupled receptor 5 (*Lgr5*), protein atonal homolog 1 (*Atoh1*), homeobox protein Nkx2.2 (*Nkx2*.*2*), LIM homeobox transcription factor 1 alpha (*Lmx1a*), and neurogenin D (*NeuroD*) were increased in Cra mice compared to GF mice irrespective of the diet (Fig. 4A), whereas ileal *Atoh1*, *Nkx2*.*2* were upregulated and *NeuroD* was downregulated (Fig. S3A) suggesting that presence of *C*. *ramosum* drives the development of colonic epithelial progenitor-cells toward secretory cells in these mice. Notch signaling maintains a balance between absorptive and secretory lineages with ATOH1 being responsible for secretory-cell development and hairy enhancer of split (HES) factors promoting the development of the absorptive lineage by inhibiting ATOH1^31^. Indeed, *Hes1* expression was downregulated in HFD-fed Cra mice (Figs 4A and S3A) indicating differentiation of intestinal stem cells toward the secretory lineage. Moreover, stimulation of SI or colonic organoids with cell lysates of *C*. *ramosum* induced the expansion of 5-HT producing ECs (for SI organoids: lysate treatment versus vehicle treatment: 6.07 ± 0.58% versus 3.56 ± 0.16% of double positive cells/nuclei, respectively, p < 0.05; for colonic organoids: lysate treatment versus vehicle treatment: 8.53 ± 1.32% versus 4.19 ± 0.36% of double positive cells/nuclei, respectively, p < 0.05, and for single positive ChA^+^ cells in SI organoids: lysates versus vehicle: 6.07 ± 0.58% versus 3.56 ± 0.16% of single positive cells/nuclei, respectively, p < 0.05; for colonic organoids: lysates versus vehicle: 8.53 ± 1.32% versus 4.47 ± 0.42% of single positive cells/nuclei, respectively, p < 0.05) (Figs 4B,C and E and S3B,C). Since intestinal crypt cells express 5-HT receptors^32,33^ and that bacterial cells probably could not directly trigger intestinal stem cells and progenitors, we speculated that *C*. *ramosum* stimulates 5-HT secretion from ECs and consequently 5-HT diffuses to the crypts where it regulates stem-cell proliferation and development. Incubation of mouse SI and colonic organoids with 10 nM 5-HT for 7 days indeed drove expansion of 5-HT-producing ECs to levels comparable with those observed in response to bacterial lysates (for SI organoids: 5-HT treatment 5.85 ± 0.45% versus vehicle treatment 3.56 ± 0.16% double positive cells/nuclei respectively, p < 0.05; for colonic organoids: 5-HT treatment 7.37 ± 0.52% versus vehicle treatment 4.19 ± 0.36% double positive cells/nuclei respectively, p < 0.05) (Fig. 4B,C and E). Furthermore, after stimulation of colonic organoids with Cra lysates, gene expression of *Tph1*, *ChA*, *Atoh1*, *Nkx2*.*2* and *Lmx1a* was increased whereas in SI organoids only 5-HT was capable of inducing upregulation of *Tph1* and *Atoh1* genes (Fig. 4D,E).

### *Clostridium ramosum* and 5-HT trigger intestinal lipid absorption

Since we demonstrated before that *C*. *ramosum* alone or as a member of a defined microbial community increased the expression of ileal genes involved in lipid absorption^4^, we investigated whether the presence of *C*. *ramosum* in monoassociated mice used in the present study or 5-HT added to intestinal cell lines also stimulates the expression of genes and proteins involved in intestinal lipid absorption.

**Figure 5:**
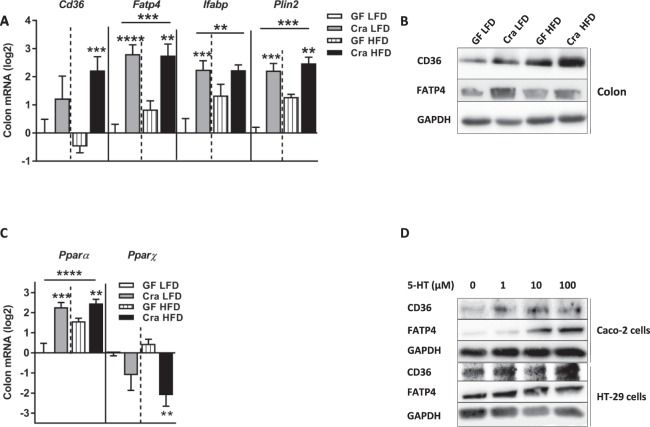
Increased expression of lipid transporters in colon is driven by 5-HT. (**A**) mRNA levels in colonic tissue of Cra and GF mice after 4 weeks on LFD or HFD determined for cluster of differentiation 36 (*Cd36*), fatty acids transport protein 4 (*Fatp4*), intestinal fatty acid binding protein (*Ifabp*) and perilipin2 (*Plin2*). (**B**) Western blot analysis with antibodies specific for CD36 (88 kDa) and FATP4 (72 kDa) in colon from indicated mice. GAPDH (36 kDa) was used as a loading control. (**C**) mRNA expression of *Pparα* and *Pparɣ* in colonic tissue derived from indicated mice. (**D**) Western blot analysis with antibodies specific for CD36 (88 kDa) and FATP4 (72 kDa) of Caco-2 and HT-29 cells derived proteins after 24 h stimulation with different concentrations of 5-HT. GAPDH (36 kDa) was used as a loading control. (**A**,**C**) Bars indicate mean ± SEM log2 fold change compared to mean of LFD-fed GF group. Asterisks above bars indicate statistical differences between Cra and GF mice for each diet group analyzed by Mann-Whitney U test. Differences among all 4 groups were analyzed by Kruskal-Wallis and Dunn’s Post-hoc tests and are indicated by lines. n = 8–13. *p < 0.05; **p < 0.01; ***p < 0.001; ****p < 0.0001. See also Fig. S4.

The mRNA levels of several genes involved in lipid transport and storage were elevated in ileum and colon of HFD-fed Cra mice compared to GF mice fed the same diet: cluster of differentiation 36 (*Cd36*), fatty acid transport protein 4 (*Fatp4*), intestinal fatty acid binding protein (*Ifabp*) and perilipin 2 (*Plin2*) (Figs 5A and S4A), suggesting increased absorption of free fatty acids in ileum and colon. The gene-expression analyses were confirmed on protein level for CD36 and FATP4 in colonic tissue (Figs 5B, S4H and S5A). Moreover, the expression of *Ppar-α* was increased in colon and ileum of HFD-fed Cra mice compared to HFD-fed GF mice while that of *Ppar-ɣ* was decreased (Figs 5B and S4B). Interestingly, ileal and colonic gene expression of *Ppar- α*, as well as ileal *Cd36*, *Fatp4* and *Ifabp* was higher in Cra mice than in GF mice, even when fed LFD, suggesting that *C*. *ramosum* influences lipid absorption in the intestine to a certain extent irrespective of the diet (Figs 5C and S4A,B).

To investigate direct effects of 5-HT on intestinal lipid absorption we stimulated two different human colorectal cell lines, namely HT-29 and Caco-2 cells, with 5-HT and observed an increase in CD36 and FATP4 protein abundance in response to increasing concentrations of 5-HT (Figs 5D and S4C,D and G). In line with these proteins abundance analyses, gene expression of *Cd36* and *Fatp4* from HT29 cells and *Fatp4* from Caco-2 cells was also elevated (Fig. S54E,F).

### Effects of *Clostridium ramosum* on lipid metabolism

**Figure 6:**
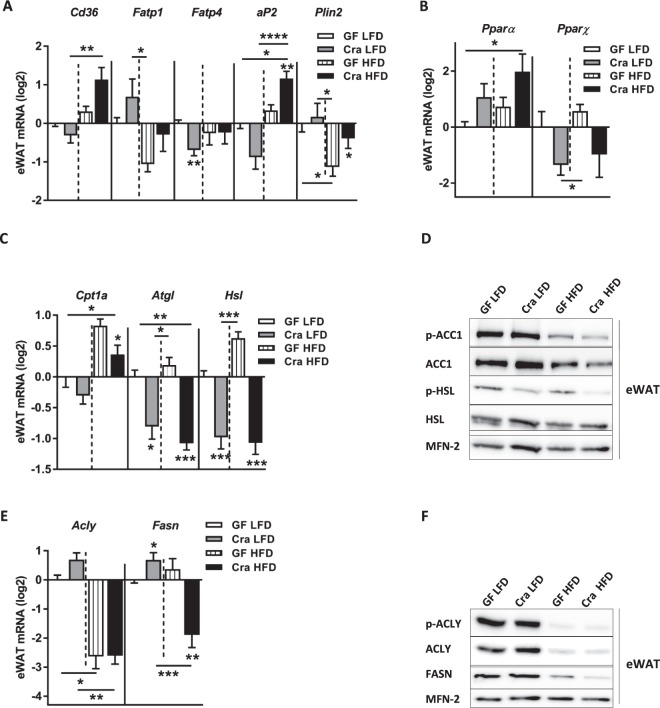
Effect of *Clostridium ramosum* on lipid metabolism in epididymal white adipose tissue (eWAT) of mice fed HFD. (**A**) mRNA expression of *Cd36*, *Fatp1*, *Fatp4*, adipocyte Protein 2 (a*P2*) and *Plin2* in eWAT of Cra and GF mice fed either LFD or HFD. (**B**,**C**) mRNA expression of *PPAR-α*, *Pparɣ*, carnitine palmitoyl transferase I (*Cpt1a*), adipose triglyceride lipase (*Atgl*) and hormone sensitive lipase (*Hsl*) in eWAT of indicated mice. (**D**) Representative Western blots of eWAT-derived proteins with antibodies specific for p-ACC1 (280 kDa), total ACC, p-HSL (81.83 kDa) or total HSL. MITOFUSIN-2 (MFN-2) (86 kDa) was used as a loading control. (**E**,**F**) mRNA and protein abundance of ATP citrate lyase (ACLY) (125 kDa) and fatty acid synthase (FASN) (273 kDa) in eWAT of indicated mice. MITOFUSIN-2 (MFN-2) (86 kDa) was used as a loading control. (**A**–**E**) Bars indicate mean ± SEM log2 fold change compared to mean of LFD-fed GF group. Asterisks above bars indicate statistical differences between Cra and GF mice for each diet group analyzed by Mann-Whitney U test. Differences among all 4 groups were analyzed by Kruskal-Wallis and Dunn’s Post-hoc tests and are indicated by lines. n = 8–13. *p < 0.05; **p < 0.01; ***p < 0.001; ****p < 0.0001. See also Fig. S5.

Recent studies reported that peripheral 5-HT regulates energy homeostasis by affecting lipid metabolism in white and brown adipose tissues^16,34^. To find out whether increased levels of 5-HT in plasma of Cra mice affected their lipid metabolism in a similar manner, we compared gene and protein expression of relevant key enzymes in adipose tissues of Cra and GF mice. First, in eWAT of Cra mice fed HFD, mRNA levels of *Cd36*, *aP2* (adipocyte Protein 2) and *Plin2* (Fig. 6A) as well as *Ppar-α* (Fig. 6B) were elevated compared to GF mice fed the same diet. Protein-abundance analysis in eWAT revealed a slight increase of CD36 in HFD-fed Cra mice versus HFD-fed GF mice (Fig. S5B,G). Neither in sWAT nor mWAT differences in gene expression of these markers were detectable (data not shown). Furthermore, gene expression of the carnitine palmitoyl transferase-1a gene (*Cpt1a*), involved in the transport of acylcarnitine into the mitochondria for β-oxidation, (Fig. 6C) was clearly reduced in eWAT of HFD-fed Cra mice compared to HFD-fed GF mice (p < 0.05). In contrast, the phosphorylation of acetyl-CoA carboxylase 1 (ACC1) was only slightly diminished in eWAT of HFD-fed Cra mice compared to GF mice on the same diet (Figs 6D and S5E). Activated (unphosphorylated) ACC1 catalyzes the carboxylation of acetyl-CoA to malonyl-CoA, which under the release of carbon dioxide undergoes condensation with acyl-CoA as catalyzed by fatty acid synthase (FASN) and leads to the generation of fatty acids of different lengths. Malonyl-CoA inactivates CPT1a and therefore, activation of ACC1 also inhibits β-oxidation. Irrespective of the diet, Cra mice displayed reduced mRNA levels of adipose triglyceride lipase (*Atgl*) and reduced gene and protein abundance of hormone sensitive lipase (HSL), which is activated via phosphorylation by cyclic AMP-dependent protein kinase (for both genes p < 0.001) (Figs 6C,D and S5B,D), suggesting the reduced lipolysis in the Cra mice. Surprisingly, HFD-fed Cra mice also showed a lower expression of genes and proteins involved in fatty acid synthesis, namely ATP citrate lyase (ACLY) and FASN, compared to LFD-fed Cra mice (p < 0.01; p < 0.001, respectively) (Figs 6E,F and S5B,E), whereas LFD-fed Cra mice exhibited increased mRNA levels of *Fasn* compared to LFD-fed GF mice (p < 0.05) (Fig. 6E). In contrast to previous studies, which reported the stimulation of BAT thermogenesis by peripheral 5-HT, we did not observe any difference in BAT thermogenesis as measured by gene and protein expression of uncoupling protein 1 (UCP1) (data not shown).

## Disscusion

Most 5-HT in the body is synthesized in the gut, particularly in large intestine, where the cell density of microbiota is highest. Several studies recently highlighted a role of intestinal bacteria in regulating 5-HT availability^24,25,35,36^. Our present work proposes a mechanistic link that helps to understand how the gut bacterium *C*. *ramosum* may promote obesity, and identifies host 5-HT as a key signaling molecule. Here we demonstrate that *C*. *ramosum* is capable of controlling 5-HT levels in plasma, intestine and eWAT preferentially by stimulating 5-HT secretion, which in turn promotes the development of 5-HT-producing ECs in the intestine and leads to further increased level of 5-HT.

After 4 weeks on a HFD, Cra mice displayed increased 5-HT levels in plasma and eWAT and a reduction of kynurenine in plasma. It is not clear if the reduction in kynurenine is a result of increased 5-HT levels since the mechanisms maintaining the balance between kynurenine and serotonin pathways are poorly understood. Increased intestinal 5-HT availability was observed in Cra HFD mice compared to GF HFD and Cra LFD mice indicating that even though *C*. *ramosum* alone increases 5-HT, the effect on host 5-HT is significantly enhanced in HFD-fed Cra mice. Surprisingly, although in eWAT of Cra HFD mice *Tph1* gene expression was increased compared to Cra LFD mice, 5-HT levels were remained unchanged. Given that 5-HT stimulates lipid absorption it may be assumed that the increased weight of eWAT of Cra HFD mice is a consequence of enhanced nutrient absorption.

The release of 5-HT has to be tightly controlled since extensive storage can favor cholera toxin-driven diarrhea and cardiac involvement^5,37,38^. We observed an increase in the degradation of 5-HT and a reduction of its further uptake into intestinal tissues in Cra mice compared to GF mice as deduced from *Maoa* and *Sert* gene-expression levels. To confirm these results we stimulated chromaffin cells as well as SI and colonic organoids with *C*. *ramosum* cell lysates or bacterial supernatants. While *C*. *ramosum* cell lysates led to an increased 5-HT release bacterial supernatants only had a modest effect. If organoids were additionally incubated with the lipid mixture, the effect exerted by Cra lysates was even enhanced suggesting that both lipids and *C*. *ramosum* contribute to 5-HT availability. The composition of the lipid mixture matched the composition of the fat component in the HFD that we used in our animal experiments: palm kern fat and sunflower oil. Other members of SIHUMI community did not display the same effect since 5-HT levels were not significantly increased after individual stimulation of ECs with lysates or supernatants of each member of the SIHUMI community.

Our data are in line with a recent report showing that spore-forming bacteria and some of their metabolites, especially deoxycholate, stimulate 5-HT release from ECs^25^. Spore-forming bacteria used in this experiment were mostly *Clostridium* species that convert cholate to the secondary bile acid deoxycholate^39,40^. However, in previous experiments with gnotobiotic mice we did not detect elevated concentrations of deoxycholate in mice harboring *C*. *ramosum* in the luminal content from small intestine, cecum or colon (data not shown). Different from the observations made in that study^25^ we found microbiota-derived soluble factors to be less effective in inducing the secretion of 5-HT than bacterial cell components. Additional experiments, which go beyond the scope of the present paper, will be required to identify those components.

Recently, it has been suggested that colonic exposure to proteins from *Escherichia coli* stimulate the gut hormone peptide YY and glucagon-like peptide-1 release from enteroendocrine cells in rats^41^. Furthermore, enterochromaffin cells are chemosensors that detect signals such as ingested chemicals and derivatives, gut microbiota metabolites and endogenous regulatory molecules and, by releasing 5-HT, they transmit the information directly to the nervous system^9^. Moreover, it was shown that *Lactobacillus rhamnosus* stimulates Nox1-dependent cell proliferation in the murine gut epithelium^42^. Using *in vitro* and *in vivo* approaches we demonstrated that *C*. *ramosum* modulates host 5-HT availability and that in turn 5-HT triggers intestinal progenitors to develop towards enteroendocrine cells through changes in expression of the key transcription factors *Hes1* and *Atoh1*. Gene-expression analyses of large intestinal tissue showed increased mRNA levels of *ChA* in Cra mice irrespective of the diet. Immunostaining and protein abundance analyses revealed that the effect of *C*. *ramosum* on EC numbers is even more pronounced in HFD-fed Cra mice compared to HFD-fed GF mice. Irrespective of the diet the transcription factors *Nkx2*.*2* and *Lmx1a* were strongly upregulated in colon of Cra mice compared to GF control animals. NKX2.2 is a homeodomain-containing transcription factor essential for the development of pancreatic islet cells. Mice with intestinal deletion of NKX2.2 in the embryo or in adults display diminished numbers of enteroendocrine cells producing 5-HT, cholecystokinin (CCK), GIP and gastrin^43,44^. LMX1A is a transcription factor that functions downstream of NKX2.2 and its intestinal deletion leads to a reduction of TPH1, the key enzyme of 5-HT synthesis^44,45^, which is in agreement with our data. Given that the stem cell marker *Lgr5* was upregulated in the colon and the majority of ChA-positive cells are also 5-HT-producting ECs we suggest that *C*. *ramosum* drives the proliferation and development of stem cells preferentially towards 5-HT-producing ECs but possibly also toward other types of secretory cells. NEUROD, expressed in pancreatic endocrine cells, the intestine, and the brain, is essential for the formation of secretin and CCK cells^45^. We observed a reduction in ileum and an increase in colon of *NeuroD* gene expression in Cra mice compared to mice devoid of this organism. Gene expression analysis of colonic organoids confirmed that treatment with Cra lysates triggers upregulation of *ChA*, *Tph1*, *Atoh1*, *Nkx2*.*2* and *Lmx1a* compared to vehicle-treated controls, whereas in SI organoids solely 5-HT induced expression of these genes. This led us to speculate that the pattern of secretory-cell development in the intestine is site-specific and possibly depends on the spatial distribution of pattern recognition receptors (PRRs). Toll-like receptors (TLRs), whose expression in small and large intestine differs^46,47^, could recognize certain components of *C*. *ramosum* lysates and thereby activate epithelial cell proliferation and development of enteroendocrine cells. TLRs are known to stimulate cell proliferation by inducing the production of amphiregulin and prostaglandin E_2_, both of which are ligands for epidermal growth factor receptor^47^. Furthermore, by sensing luminal bacteria, intestinal myeloid cells alter epithelial-cell differentiation preferentially in the colon, where the density of macrophages is higher than in the small intestine^48^.

Endogenous 5-HT promotes growth and turnover of the intestinal mucosal epithelium but these processes appear to be mediated by neuronal, rather than mucosal 5-HT^32^. However, we observed increased 5-HT secretion from chromaffin cells upon stimulation with heat killed *C*. *ramosum* cells indicating that mucosal 5-HT secretion is stimulated by this bacterium, which does not exclude the involvement of neuronal 5-HT *in vivo*.

Genetic and pharmacological (LP533401) inhibition of TPH1 protects HFD-fed mice from obesity, chronic low-grade inflammation, fatty liver disease and insulin resistance via activation of UCP1-mediated thermogenesis^16^. On chow diet TPH1 knockout mice develop diabetes and are mildly insulin-resistant^49^. Furthermore, another TPH1 pharmacological inhibitor, *p*-chlorophenyl alanine, inhibits lipogenesis in eWAT and induces browning in inguinal WAT of mice^34^.

In our experiments monoassociation with *C*. *ramosum* enhanced HFD-induced obesity, as may be deduced from increased body and eWAT weight, plasma 5-HT level and intestinal expression of lipid transporters after high-fat diet feeding, all of which is consistent with previous results^4^. The digested lipids from the lumen are taken up by enterocytes mostly by transporters. CD36 and FATP4 facilitate fatty-acid uptake across plasma membranes^50,51^, whereas in the cytosol IFABP transport fatty acids to cell organelles and coordinate lipid responses in cells^52^. PLIN2, a member of the PAT protein family, is a crucial regulator of lipid storage in the lipid droplets in adipose and non-adipose tissues^53,54^. Activation of peroxisome proliferator-activated receptors (PPARs) transcription factors, especially PPAR-α and PPAR-γ results in the induction of genes involved in lipid and glucose metabolism^55,56^. Although PPAR isotypes share similarities in function and mechanism of action, they display important tissue-specific physiologic differences^57^. In intestine, PPAR-α mostly mediates the absorption of nutrients^55,58^ whereas in the liver, heart and adipose tissue is a major activator of fatty acid oxidation pathways^59^. PPAR-γ has been shown to be down-regulated in the intestine by a number of bacterial pathogens including *Helicobacter pylori*, *Mycobacterium tuberculosis* and *Salmonella enterica* serovar Typhimurium, aggravating infections^60^. Since the association of GF mice with *C*. *ramosum* does not cause intestinal inflammation (data not shown), the detected reduction in *Ppar-ɣ* gene expression levels suggest that *C*. *ramosum* may interact with epithelial cells via the similar route, while inducing a non-inflammatory response. PPAR-γ is a master regulator of adipocyte differentiation, moreover it enhances fatty acid uptake^59,61^ and maintains the thermogenic capacity of brown adipocytes^62^.

In detail, we observed upregulation of CD36, FATP4, PLIN2 and *Ppar-α* in ileum and colon of Cra mice compared to GF mice indicating that *C*. *ramosum* promotes absorption of fatty acids in the intestine and that the process is triggered by *Ppar-α* activation. Contradictory to the literature, in eWAT of Cra mice we detected increased *Ppar-α* and reduced *Ppar-ɣ* gene expression suggesting that this transcription factors have broader repertoire of functions than previously thought.

By incubating HT-29 and Caco-2 cells with 5-HT we confirmed that 5-HT stimulates expression of major proteins involved in intestinal translocation of fatty acids. Therefore, we suggest as a possible link to obesity an increased lipid absorption, which is driven by *C*. *ramosum* presence in the gut. However, is there causal relationship between obesogenic phenotype of Cra mice and increased 5-HT availability remains open question and needs further investigations.

It was reported that 5-HT promotes gluconeogenesis in liver and suppresses adaptive thermogenesis and glucose uptake in BAT^16,22,34^, but we observed no differences in these processes between the Cra and GF mice. However, we detected increased expression of markers of lipid absorption and reduced expression of markers of lipolysis, β-oxidation and lipogenesis in eWAT of HFD-fed Cra mice. A recent study using 3T3-L1 adipocytes reported that 5-HT and 5-HT receptor agonist treatment increased lipid accumulation and suppressed lipolysis^34^, which is in conflict with a study showing that 5-HT favors lipolysis as an adaptation to fasting^22^. Such apparently contradictory results may be due to differences in the genetic background of the mice and in microbiota composition caused by differences in the diets.

By analyzing ATGL and HSL mRNA and protein abundance we observed a significant downregulation of lipolysis in eWAT of mice harboring *C*. *ramosum*. Furthermore, β-oxidation of fatty acids was reduced in eWAT from Cra mice fed a HFD. On the other hand, reduced ACLY and FASN suggested suppressed lipogenesis, which is in conflict with the published data^34^. However, this process does not necessarily depend on the gut microbiota since ACLY was downregulated in both HFD-fed groups of mice. Major differences between our and the above mentioned studies are host genetics and duration of the treatment (4 weeks versus 12 weeks of feeding, respectively) which could explain the discrepancies between the results.

Taken together, these data indicate that *C*. *ramosum* promotes 5-HT secretion from ECs. Subsequently 5-HT diffuses to cells located in the intestinal crypts and programs differentiation of colonic stem progenitors towards the secretory 5-HT-producing lineage. The resulting elevated 5-HT levels could possibly upregulate the expression of major proteins involved in intestinal fatty acid absorption. Therefore, we propose that increased 5-HT levels in intestinal tissues triggered by the presence of *C*. *ramosum* may promote the nutrient absorption and therefore contribute to obesity development.

## Material and Methods

### Mice and experimental setup

Germ-free male C3H/HeOuJ mice were obtained from the gnotobiotic animal facility of the German Institute of Human Nutrition, Potsdam-Rehbruecke, Germany. The mice were maintained in positive pressure isolators (Metall & Plastik, Radolfzell, Germany) under a 12 h light-dark cycle at 22 ± 2 °C and 55 ± 5% air humidity. All mice were kept individually in polycarbonate cages on irradiated wood chips (25–50 kGy). The mice had free access to irradiated standard chow (Altromin fortified type 1310, Altromin, Lage, Germany) and autoclaved water. The animal experiments were approved by the animal welfare committee of the state of Brandenburg (approval no. V3–2347–10–2011, V3-2347-02-13). Germ-free male mice were monoassociated with *C*. *ramosum* DSM 1402 (Cra) at the age of 6 weeks. Twelve-week old germ-free and Cra mice were either fed ad libitum a semi-synthetic LFD or HFD (see Supplementary Table 1). Body weight was determined weekly. After 4 weeks of dietary intervention, body composition was determined by quantitative magnetic resonance spectroscopy (MQ10, Bruker Minispec, Houston, TX, USA), the non-fasted mice were anaesthetized and blood was taken from the retrobulbar plexus, followed by cervical dislocation of the mice. Liver and adipose tissues were weighed. Mucosa was scraped from ileum and colon. All tissues were frozen in liquid nitrogen and stored at −80 °C.

All methods were performed in accordance with the relevant guidelines and regulations.

### Immunofluorescence

Paraffin-embedded tissue sections were deparaffinized and subjected to antigen retrieval for 10 min in 10 mM sodium citrate, pH 6.0 and incubated overnight at 4 °C with primary antibodies (see Supplementary Table 4). At the following day the slides were incubated for 2 h at room temperature (RT) with secondary antibodies (see Supplementary Table 4) and mounted with 4′,6-diamidino-2-phenylindole (DAPI) -containing mounting solution (Vector Laboratories, Lörrach, Germany) for counterstaining of nuclei. Samples were imaged with an Axio Imager Z1 (Carl Zeiss, Jena, Germany) and 5-HT and ChA positively-stained cells were scored blindly, normalized to total cell number using ImageJ software (NIH, Bethesda, ML, USA) and presented as percentage of total cell number (cells/nuclei (%)).

### 5-HT measurement

Serotonin levels were detected in supernatants of tissue homogenates and cell supernatants by ELISA (enzyme-linked immunosorbent assay) according to the manufacturer’s instructions (DLD Diagnostika, Hamburg, Germany). Serotonin values were normalized to total protein content as determined with the Bradford method^63^.

### RNA isolation and RT-PCR

RNA from intestinal mucosa and adipose tissue was isolated with the peqGOLD TriFast reagent containing phenol (Peqlab, Erlangen, Germany). After adding the chloroform and centrifugation for 5 minutes at 12000 × g the mixture separates into the lower red phenol-chloroform phase containing proteins, the interphase containing proteins and DNA and the colorless upper aqueous phase containing RNA. Genomic DNA was removed using the Ambion Turbo DNA-free kit (Life Technologies, Darmstadt, Germany). Integrity of RNA was verified by running aliquots on agarose gels stained with ethidium bromide. Complementary DNA was synthesized from 1 µg of total RNA with the Revert AidH Minus first-strand cDNA synthesis kit (Thermo Scientific, Braunschweig, Germany). Quantitative PCR was performed with the Applied Biosystems 7900 HT fast real-time PCR system (Life Technologies, Darmstadt, Germany). The reaction mixture of 5 µl contained 2.5 µl Power SYBR green PCR master mix, 3 µM gene specific primers (see Supplementary Table 2) and a cDNA amount corresponding to 5 ng of RNA. Data were analyzed by the ΔΔCt method using *Hprt* (Hypoxanthine-guanine phosphoribosyl transferase) for normalization and presented as log2 fold change.

### Western blot

Proteins from tissues were obtained from the lower phenol-chloroform phase formed during phenol RNA extraction (see above) or isolated from cells and quantified with the Bradford method. Briefly, proteins are precipitated with acetone, washed 3 times with a solution of 0.3 M guanidinium hydrochloride (Sigma-Aldrich, Munich, Germany) in 95% ethanol and dried under vacuum. Protein extracts (30 µg) were electrophoresed and then blotted following standard procedures^64,65^. Primary antibodies were specific for CHA, CD36, p-ACC1, total ACC, p-HSL, total HSL, p-ACLY, total ACLY, FASN, GAPDH and MITOFUSIN-2 (see Supplementary Table 3). Anti-rabbit and anti-mouse IgG-HRP (horseradish peroxidase) were used as secondary antibodies. Blots were incubated for 5 min with ECL solution (GE Healthcare, Munich, Germany), containing an HRP-substrate. Emission of a chemiluminescence signal was detected with a computer-assisted camera system (PeqLab). If required blots were stripped with 10 ml Stripping solution (Thermo Scientific, Braunschweig, Germany), washed with TBS-T (20 mM Tris pH 7.5, 150 mM NaCl and 0.1% Tween 20) and reused.

### Metabolome analysis

The metabolome analysis were carried out with the Absolute*IDQ*® p180 Kit (Biocrates Life Science AG, Innsbruck, Austria) using a liquid chromatography–mass spectrometry method. The integrated Met*IDQ* Software (Biocrates Life Science AG, Innsbruck, Austria) streamlines data analysis by automated calculation of metabolite concentrations. Quantification of analytes utilizes stable isotope-labelled or chemically homologous internal standards. Controls are included for 3 different concentration levels. For calibration the Kit contains a calibrator mix with 7 different concentrations. The measurements were carried out by an AB Sciex qtrap 5500 mass spectrometer via Electrospray ionization (ESI) by Multi Reaction Monitoring (MRM) mode for high specificity and sensitivity. 158 MRM pairs were measured in positive ion mode (13 IS) and 2 MRM pairs were measured in negative mode (1 IS). In the LC-MS/MS method 65 MRM pairs were measured only in positive mode (25 IS).

### Production of bacterial lysates and bacterial supernatants

*Clostridium ramosum* DSM 1402 was cultured at 37 °C under strictly anoxic conditions to late exponential growth phase in a mixture of Brain Heart Infusion Broth (BHI) and fatty acid medium (YCFA) (BHI: YCFA, 50:50, v/v). BHI (Roth) was supplemented with 5 g/l yeast extract (Roth), 5 mg/l haemin (Sigma-Aldrich, Munich, Germany), 0.5 g/l cysteine hydrochloride (Sigma-Aldrich, Munich, Germany) and 0.9 mg/l resazurin (Thermo Scientific, Braunschweig, Germany). The composition of YCFA is listed in Supplementary Table 4. *Anaerostipes caccae* DSM 14662, *Bacteroides thetaiotaomicron* DSM 2079, *Bifidobacterium longum* NCC 2705, *Blautia producta* DSM 2950, *Clostridium butyricum* DSM 10702, *Escherichia* coli K-12 MG1655 and *Lactobacillus plantarum* DSM 20174 were cultured at 37 °C under strictly anoxic conditions to late exponential growth phase only in BHI. Supernatants were harvested after centrifugation (10 min, 8000 × g) and stored at −20 °C. Bacterial cells were washed once in reduced phosphate buffered saline (rPBS in g/l: NaCl 8.5; KH_2_PO_4_ 0.3; Na_2_HPO_4_ 0.6; bacto peptone 0.1; cysteine × HCl × H_2_O 0.25; resazurine 0.001; pH 7). For the preparation of lysates, bacterial cells were incubated in a thermal block unit at 96 °C for 30 min. Afterwards, the heat-inactivated cells were centrifuged (10 min, 800 × g) and the pellets of all 8 bacteria were resuspended in BHI/YCFA medium to adjust 10^8^ cells per ml. The absence of viable cells was confirmed by plating the suspension on Columbia blood agar (Biomérieux, France). Bacterial supernatants were filtered through a 0.2 μm syringe filter to remove remaining intact bacteria.

### Cell culture

The rat pancreas cell line RIN14B (ATCC Cat# CRL-2059, RRID: CVCL_3583) was cultured in humidified incubators at 37 °C, 5% CO_2_ in RPMI medium 1640 supplemented with 10% fetal bovine serum, 25 mM glucose, 1 mM sodium pyruvate, 1 mM Hepes buffer pH 7.4 and 10 units/ml penicillin/streptomycin (all from PAN-Biotech, Aidenbach, Germany). The cells were seeded into 24-well plates (2 × 10^5^ cells/well), grown to sub-confluency and washed with Hanks’ Balanced Salt solution (HBSS) containing 0.2% BSA and 2 µM fluoxetine (Tocris, Minneapolis, USA) and incubated for 1 h with bacterial lysates (final concentration 10^7^/ml) or supernatants derived from bacterial cultures. Positive control cells were treated with 15 µM ionomycin (Sigma-Aldrich, Munich, Germany), in vehicle (HBSS). After incubation, supernatants were collected, centrifuged at 6000 × g for 5 min and frozen for downstream 5-HT assays. Remaining adherent RIN14B cells were lysed in Trizol (Thermo Scientific, Braunschweig, Germany) for downstream RNA isolation, cDNA synthesis and qRT-PCR as described above. Cytotoxic effects were excluded via neutral red cytotoxicity assays performed with the *in vitro* toxicology kit (Sigma-Aldrich, Munich, Germany). HT29 cells (ATCC Cat# HTB-38, RRID: CVCL_0320) and Caco-2 cells (ATCC Cat# HTB-37, RRID: CVCL_0025) were grown in the same medium as RIN14B cells and seeded into 6-well plates (10^5^ cells/well). Sub-confluent cells were treated with different concentrations of serotonin (0, 1, 10 and 100 µM, Sigma-Aldrich, Munich, Germany) or PBS as control. After 24 h, cells were harvested for RNA isolation or protein analysis with phosphate buffered saline (PBS) (37 mM NaCl, 2.7 mM KCl, 4.3 mM Na_2_HPO_4_, 1.47 mM KH_2_PO_4_; pH 7.4) without Ca^2+^ and Mg^2+^ with 5 mM EDTA (PAN-Biotech, Aidenbach, Germany) for 20 min on ice and afterwards resuspended in 200 µl lysis buffer (50 mM Tris-HCl pH 7.5, 150 mM NaCl, 0.5 % NP-40, 50 mM NaF, 1x protease inhibitor Complete Mini (1 tablet/10 ml of buffer), 1x phosphatase inhibitor PhosSTOP (1 tablet/10 ml of buffer) (both Roche, Basel, Switzerland) and 1 mM Dithiothreitol (Sigma-Aldrich, Munich, Germany).

### Organoid culture

C57BL/6 mice were maintained and killed by cervical dislocation according to the animal law. Crypts from small and large intestines were isolated from 10-week old C57BL/6 male mice (three mice per group) as described previously^66^. In brief, crypts were isolated by incubating 0.5 cm long gut pieces in isolation buffer (PBS [37 mM NaCl, 2.7 mM KCl, 4.3 mM Na_2_HPO_4_, 1.47 mM KH_2_PO_4_; pH 7.4] without Ca^2+^ and Mg^2+^ with 2 mM EDTA) (PAN-Biotech, Aidenbach, Germany) for 30 min at 4 °C. Isolated crypts were embedded in matrigel (growth factor-reduced, phenol red-free, BD Biosciences, Heidelberg, Germany) and seeded into 48-well plates (20 µl of matrigel per well). The matrigel was polymerized for 15 min at 37 °C, and 250 μl IntestiCult Organoid Growth Medium (Stem cell Technologies, Cologne, Germany) was added per well. Fresh medium was added at day 4 and organoids were passaged after 7 days with a 1:4 split ratio. Through mechanical shearing organoids were disrupted into single-cell suspensions and mixed with bacterial lysates (final concertation 10^7^/ml), supernatants derived from bacterial cultures or 5-HT (10 nM) and embedded in matrigel in 48-well plates for gene expression analysis or in 8-well µ-Slides (Ibidi, Planegg, Germany) for immunofluorescence staining. For 5-HT ELISA measurement colonic organoids are stimulated with 2% Lipid Mixture 1 ((2 μg/ml arachidonic and 10 μg/ml each linoleic, linolenic, myristic, oleic, palmitic and stearic), (Sigma-Aldrich, Munich, Germany) in parallel with bacterial stimulation for 1 hour and supernatants were collected. Samples for RNA isolation were obtained as described above. Fluorescence microscopy was performed using a laser confocal microscope (Carl Zeiss, Jena, Germany).

## Supplementary information

Supplementary Dataset 1

## Data Availability

The datasets generated during and/or analyzed during the current study are available from the corresponding author on request.
