# Supplementary material for: Clostridium ramosum regulates enterochromaffin cell development and serotonin release
Source: Sci Rep. 2019 Feb 4;9:1177. doi: 10.1038/s41598-018-38018-z (PMC6362283; doi:10.1038/s41598-018-38018-z)
Supplement: Supplementary file 1 — Supplementary Dataset 1 [file 41598_2018_38018_MOESM1_ESM.pdf]

SUPPLEMENTARY MATERIAL

Clostridium ramosum regulates enterochromaffin cell development and serotonin release

Authors: Ana D. Mandić,[1] Anni Woting,[1] Tina Jaenicke,[1] Anika Sander,[1] Wiebke Sabrowski,[1] Ulrike Rolle-Kampcyk,[2] Martin von Bergen,[2,3] and Michael Blaut[1]

Author affiliations:

[1]Department of Gastrointestinal Microbiology, German Institute of Human Nutrition Potsdam-Rehbruecke, Nuthetal, Germany

[2]Department of Molecular Systems Biology, Helmholtz Centre for Enviromental Research – UFZ, Germany

[3]Institute of Biochemistry, Faculty of Life Sciences, University of Leipzig, Leipzig, Germany

SUPPLEMENTARY FIGURE LEGENDS

Figure S1. Related to Figure 1. Impact of *Clostridium ramosum* on body composition. (A) Total body fat mass of Cra and GF mice after 4 weeks of either LFD or HFD feeding. (B, C, D & F) mWAT, sWAT, BAT and liver relative weight after 4 weeks on LFD or HFD. (E) Blood glucose values of indicated mice. (G) Length of small intestine from indicated mice. (H) Weight of full cecum from indicated mice. (I) Percentage of total body lean mass from

indicated mice. (J) Weight change after 4 weeks of either LFD or HFD feeding. .* p< 0.05; ** p< 0.01; *** p< 0.001 for Cra LFD versus Cra HFD; ### p< 0.001 for GF HFD versus Cra HFD. Means ± SEM of n = 8 – 13 per group are shown. (A –I) Asterisks above bars indicate statistical differences between Cra and GF mice with respect to the LFD or HFD diet group analyzed by Mann-Whitney U test. Differences among all 4 groups were analyzed by Kruskal-Wallis and Dunn's Post-hoc tests and are indicated by lines.* p< 0.05; ** p< 0.01; *** p< 0.001

Figure S2. Related to Figure 2. *Clostridium ramosum* regulates peripheral 5-HT availability. (A & B) Levels of plasma serotonin and tryptophan from Cra and GF mice fed either LFD or HFD. (C) Ratio of plasma kynurenine and tryptophan concentrations from indicated mice. (D) mRNA expression in ileal tissue Cra and GF mice fed either LFD or HFD for 4 weeks determined for *ChA*, *Tph1*, *Sert* and *Maoa*. Means ± SEM of n = 8 – 13 per group are shown. Asterisks above bars indicate statistical differences between Cra and GF mice with respect to the LFD or HFD diet group as analyzed by Mann-Whitney U test. Differences among all 4 groups were analyzed by Kruskal-Wallis and Dunn's Post-hoc tests and are indicated by lines. (E) Densitometry analysis of proteins isolated from indicated mice for ChA. Mean ± SEM of relative density normalized to housekeeping protein and LFD-fed GF mice. n = 5 – 8. (F) Full length western blot protein analysis cropped from different parts of the same gel with a ChA-specific antibody (49 kDa) applied to colonic mucosa isolated from indicated mice. GAPDH (36 kDa) was used as a loading control. Representative samples presented in Figure 2C are indicated by lines.* p< 0.05; ** p< 0.01; *** p< 0.001; **** p< 0.0001.

Figure S3. Related to Figure 4. *Clostridium ramosum* induces expansion of enterochromaffin cells. (A) Ileal mRNA levels of *Lgr5*, *Atoh1*, *Nkx2.2*, *Lmx1a*, *NeuroD* and *Hes1* in Cra and GF mice fed either LFD or HFD. Bars indicate mean ± SEM log2 fold

change compared to mean LFD-fed GF group. (B & C) Quantification of ChA positive colonic ECs from small intestinal and colonic organoids. Means ± SEM of n = 5 – 12 per group are shown. (D & E) mRNA levels of *Sert* in small intestinal and colonic organoids. Bars indicate mean ± SEM log2 fold change compared to mean of vehicle-treated controls (n = 3 – 4). Asterisks above bars indicate statistical differences between Cra and GF mice in respect to the diet group as analyzed by Mann-Whitney U test. Differences among all 4 groups were analyzed by Kruskal-Wallis and Dunn's Post-hoc tests and are indicated by lines. * $p< 0.05$; ** $p< 0.01$; *** $p< 0.001$.

Figure S4. Related to Figure 5. Increased intestinal lipid absorption is driven by 5-HT. (A) mRNA expression of *Cd36, Fatp4, Ifabp* and *Plin2* in ileum derived from Cra and GF mice after 4 weeks on HFD and LFD. (B) mRNA expression of *Ppara* and *Pparγ* in ileum of indicated mice. Bars indicate mean ± SEM log2 fold change compared to mean of LFD-fed GF group. Asterisks above bars indicate statistical differences between Cra and GF mice for each diet group, which were analyzed by Mann-Whitney U test. Differences among all 4 groups were analyzed by Kruskal-Wallis and Dunn's Post-hoc tests and are indicated by lines. n = 8 – 13. * $p< 0.05$; ** $p< 0.01$; *** $p< 0.001$. **** $p< 0.0001$ (C & D) Densitometry analysis of proteins isolated from HT-29 and Caco-2 cells for CD36 and FATP4. Mean ± SEM of relative density normalized to GAPDH and control samples. n = 4 – 6. (E & F) mRNA expression of *Cd36* and *Fatp4* in HT-29 and Caco-2 cells after 24 h treatment with different concentrations of 5-HT. Means ± SEM of n = 6 – 8 per group are shown. (G) Full length western blot protein analysis cropped from different parts of the same gel with a CD36-specific antibody (88 kDa) and FATP4 (72 kDa) of proteins derived from and Caco-2 cells (upper panel) and HT-29 cells (lower panel). GAPDH (36 kDa) was used as a loading control. Representative samples presented in Figure 5D are indicated by lines. (H) Full length western blot protein analysis cropped from different parts of the same gel with a CD36-

specific antibody (88 kDa) and FATP4 (72 kDa) applied to colonic mucosa isolated from indicated mice. GAPDH (36 kDa) was used as a loading control. Representative samples presented in Figure 5B are indicated by lines. Differences among all 4 groups were analyzed by one-way Anova and Tukey's multiple comparison tests and are indicated by lines.* $p < 0.05$; ** $p < 0.01$; *** $p < 0.001$

Figure S5. Related to Figure 6. Increased expression of lipid transporters in Cra mice fed HFD. (A) Densitometric analysis of colonic mucosa protein abundance for CD36 and FATP4. Mean ± SEM of relative density normalized to GAPDH and LFD-fed GF. n = 5 – 6. (B) Densitometry analysis of eWAT proteins isolated from indicated mice for CD36, p-ACC1, p-HSL, p-ACLY and FASN. Mean ± SEM of relative density normalized to housekeeping protein and LFD-fed GF mice. n = 5 – 6. Differences among all 4 groups were analyzed by Kruskal-Wallis and Dunn's Post-hoc tests and are indicated by lines. n = 8 – 13. * $p < 0.05$; ** $p < 0.01$; *** $p < 0.001$. (C) Full length western blot protein analysis cropped from different parts of the same gel with CD36-specific antibody (88 kDa) of eWAT proteins isolated from indicated mice. MITOFUSIN-2 (MFN-2) (86 kDa) was used as a loading control. Representative samples are indicated by lines. (D) Full length western blot protein analysis cropped from different parts of the same gel with a p-HSL (81.83 kDa) or total HSL (88 kDa) of eWAT proteins isolated from indicated mice. MITOFUSIN-2 (MFN-2) (86 kDa) was used as a loading control. Representative samples presented in Figure 6D are indicated by lines. (E) Full length western blot protein analysis cropped from different parts of the same gel with a p-ACC1 (280 kDa), total ACC, p-ACLY (125 kDa), total ACLY or FASN (273 kDa) of eWAT proteins isolated from indicated mice. MITOFUSIN-2 (MFN-2) (86 kDa) was used as a loading control. Representative samples presented in Figure 6D & F are indicated by lines.

SUPPLEMENTARY TABLES

Supplementary table 1 Composition of the semi-synthetic low-fat diet (LFD) and the high-fat diet (HFD).

Ingredient	HFD (g/100 g)	LFD (g/100 g)
Casein	27	22
Wheat starch	15	38
Maltodextrin	14	14
Sucrose	10	10
Palm kernel fat (Palmin)	11	2
Sunflower oil	11	2
Cellulose	5	5
Mineral mixture	5	5
Vitamin mixture	2	2
Energy (kJ/g)[a]	20.6	17.3
Protein (energy %)	22.7	23.8
Carbohydrate (energy %)	32.6	65.7
Fat (energy %)	44.7	10.5

[a]Determined by bomb calorimetry.

Supplementary table 2.

RT-qPCR primers

**Target gene**	**Primer name**	**Primer sequence in 5' – 3' orientation**
Hprt	Forward	CAG TCC CAG CGT CGT GAT TA
	Reverse	AGC AAG TCT TTC AGT CCT GTC
Gapdh	Forward	CAACTTTGTCAAGCTCATTTCC
	Reverse	TCCAGGGTTTCTTACTCCTTG
Tph1	Forward	CAGCAAGGACGGGATCAACT
	Reverse	CACTCTCCCTCTTTCGGAGG
Sert	Forward	CAA AA CCA AGA ACC AAG AG
	Reverse	CAT AGC CAA TGA CAG ACAG
Maoa	Forward	GGAGAAGCCCAGTATCACAGG
	Reverse	GAACCAAGACATTAATTTTGTATTCTGAC

5-Ht2a	Forward	AGCTGCAGAATGCCACCAACTAT
	Reverse	GGGATTGGCATGGATATACCTA
5-Ht2b	Forward	AAATAAGCCACCTCAACGCCT
	Reverse	TCCCGAAATGTCTTATTGAAGA
ChA	Forward	ACTTCCATGCAGGCTACAAAGC
	Reverse	CTCTGTCTTTCCATCTCCATCCA
Lgr5	Forward	CAG GCC GTC TGT GAT CAG TT
	Reverse	GCA GCC TGA CAA ACT GGG TA
Atoh1	Forward	GAG TGG GCT GAG GTA AAA GAG T
	Reverse	GGTCGGTGCTATCCAGGAG
Nkx2.2	Forward	CCAACAGGAGCGGGACAT
	Reverse	CAAACACAAATACAAACCGATTGC
Lmx1a	Forward	AACCAGCGAGCCAAGATGAA
	Reverse	CCCGCATTCCCACTACCATT
Hes1	Forward	TCA ACACGACACCGGACAAAC C
	Reverse	GGTACTTCCCCAACACGCTCG
NeuroD	Forward	GGA GTA GGG ATG CAC CGG GAA
	Reverse	CTT GGC CAA GAA CTA CAT CTG G
Ppara	Forward	TGG CAA AGT CTT AGT GCC AGA
	Reverse	TCA CTA GGT CAC ACA GCC TCT
Pparɣ	Forward	TGC CAA AAA TAT CCC TGG TT
	Reverse	GGC GGT CTC CAC TGA GAA TA
Cpt1a	Forward	CCA AAC CCA CCA GGC TAC A
	Reverse	GCA CTG CTT AGG GAT GTC TCT ATG
Atgl	Forward	AAC ACC AGC ATC CAG TTC AA
	Reverse	GGT TCA GTA GGC CAT TCC TC
Hsl	Forward	GCT TGG TTC AAC TGG AGA GC
	Reverse	TGC CTC TGT CCC TGA ATA GG
Cd36	Forward	CCA AGC TAT TGC GAC ATG AT
	Reverse	ACA GCG TAG ATA GAC CTG CAA A
Plin2	Forward	GTG TGT GAG ATG GCC GAGAA
	Reverse	AAC AAT CTC GGA CGT TGG CT
Fabp4 (Ap2)	Forward	ACA CCG AGA TTT CCT TCA AAC TG
	Reverse	CCA TCT AGG GTT ATG ATG CTC TTC A
Fatp4	Forward	TTC ATC AAG ACG GTC AGG AG
	Reverse	ACC ATT GAA GCA AAC AGC AG
Fabp2	Forward	GTT GTG TTT GAG CTC GGT GT
	Reverse	AGC AAT CAG CTC CTT TCC AT
Rat *Gapdh*	Forward	GTCGGTGTGAACGGATTTG
	Reverse	TGG AAG ATG GTG ATG GGT TT
Rat *Tph1*	Forward	CAA GGA GAA CAA AGA CCA TTC

	Reverse	CGC AGT CCA CAA AAA TCT CA
Rat *Sert*	Forward	ATG GAG ACC AGC ACA CCC TTG A
	Reverse	GTG GGG ACA CCC TTC TGT A
Human *Hprt*	Forward	TGG CGT CGT GAT TAG TGA TG
	Reverse	GGC CTC CCA TCT CCT TCA T
Human *Cd36*	Forward	TCT TTC CTG CAG CCC AAT G
	Reverse	AGC CTC TGT TCC AAC TGA TAG TGA
Human *Fatp4*	Forward	CTG GG TGG CTG CCT GAT TAT
	Reverse	AGG CAG AGT TTA TTG GCC CC

Supplementary table 3.

Primary antibodies

**Antigen**	**Source**	**Indentifier**
5-HT	Abcam	Cat# ab6336 RRID: AB_449517
ACC1	Cell Signaling	Cat# 4190S RRID: AB_10547752
CD36	R&D systems	Cat# MAB2519 RRID:AB_2072634
CHA	Abcam	Cat# ab15160 RRID: AB_301704
FASN	Cell Signaling	Cat# 3189 RRID: AB_2100798
FATP4	Abcam	Cat# ab199719 RRID: AB_2716563
GAPDH	Ambion	Cat# AM4300 RRID: AB_437392
HSL	Cell Signaling	Cat# 4107 RRID: AB_2296900
MITOFUSIN-2	Cell Signaling	Cat# 9482 RRID: AB_2716838
p-ACC1	Cell Signaling	Cat# 11818 RRID: AB_2687505
p-HSL	Cell Signaling	Cat# 4137 RRID: AB_2135498

Secondary antibodies

**Antigen, labelling**	**Source**	**Indentifier**
Mouse IgG -HRP	Cell Signaling	Cat# 7076 RRID: AB_330924
Rabbit IgG -Alexa 594	Thermo Fisher	Cat# A-11007 RRID: AB_10561522
Rabbit IgG-HRP	Cell Signaling	Cat# 7074 RRID: AB_2099233
Rat IgG-HRP	Cell Signaling	Cat# 7077 RRID: AB_10694715
Rat IgG-Alexa 488	Thermo Fisher	Cat# A-11034 RRID: AB_2576217

Supplementary table 4.

Preparation of medium 496 YCFA GSC

Composition (amount per liter H2Odd)

	10.0 g
Cellobiose	2.0 g
Glucose	2.0 g
Haemin solution (0.5 g/l)	20 ml
L-cysteine x HCl (added after boiling)	1.445 g
Maltose	2.0 g
Mineral solution I	150 ml
Mineral solution II	150 ml
NaHCO3	4.0 g
Resazurin	1 ml
VFA mix	3.1 ml
Vitamin solution	1ml
Yeast extract	2.5 g

Mineral solution I (g/l)

K2HPO4	3g

Mineral solution II (g/l)

KH2PO4	3 g
(NH4)2SO4	6 g
NaCl	6 g
MgSO4 x 7 H2O	1.22 g
CaCl2 x 2 H2O	0.92 g

VFA mix

Acetic acid	17 ml
Iso-butyric acid	1 ml
Iso-valeric acid	1 ml
n-Valeric acid	1 ml
Propionic acid	6 ml

Vitamin solution (mg/l)

4-aminobenzoic acid	30 mg
Biotin	10 mg
Cobalamin	10 mg
Folic acid	50 mg
Pyridoxamine	150 mg

Statistics

Data are expressed as means ± standard errors of the means (SEM). Statistical significance was analyzed by Mann Whitney U test for comparison of two groups and by Kruskal-Wallis test followed by Dunn's post hoc test for the comparison of multiple groups or one-way Anova and Tukey's multiple comparison tests where indicated. Statistical analyses were performed using GraphPad Prism 6.0 (GraphPad Software, Inc., La Jolla, CA).

Figure S1

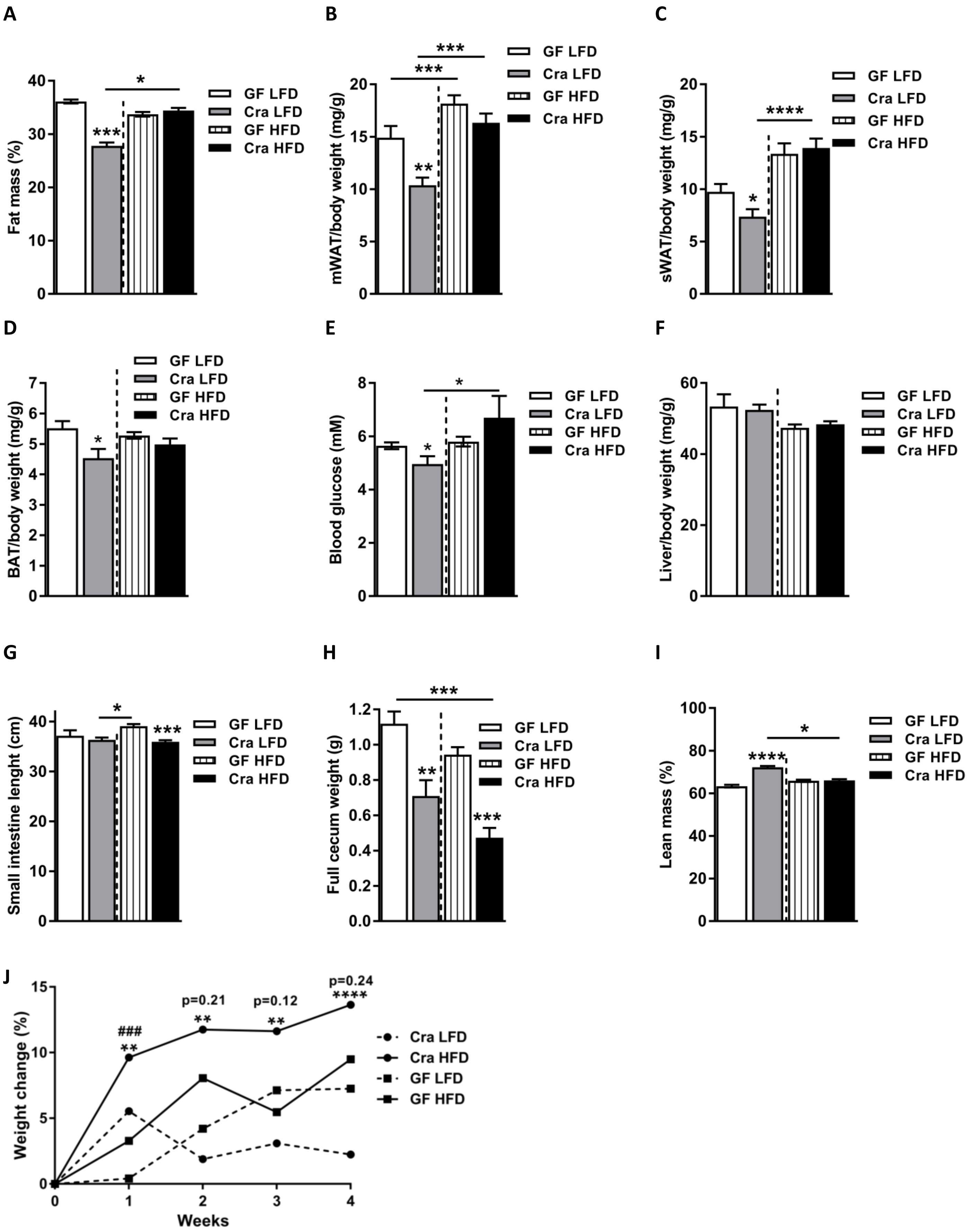

A
Fat mass (%)
GF LFD
Cra LFD
GF HFD
Cra HFD
B
mWAT/body weight (mg/g)
C
sWAT/body weight (mg/g)
D
BAT/body weight (mg/g)
E
Blood glucose (mM)
F
Liver/body weight (mg/g)
G
Small intestine lenght (cm)
H
Full cecum weight (g)
I
Lean mass (%)
J
Weight change (%)
Weeks
p=0.21
p=0.12
p=0.24
###

Figure S2

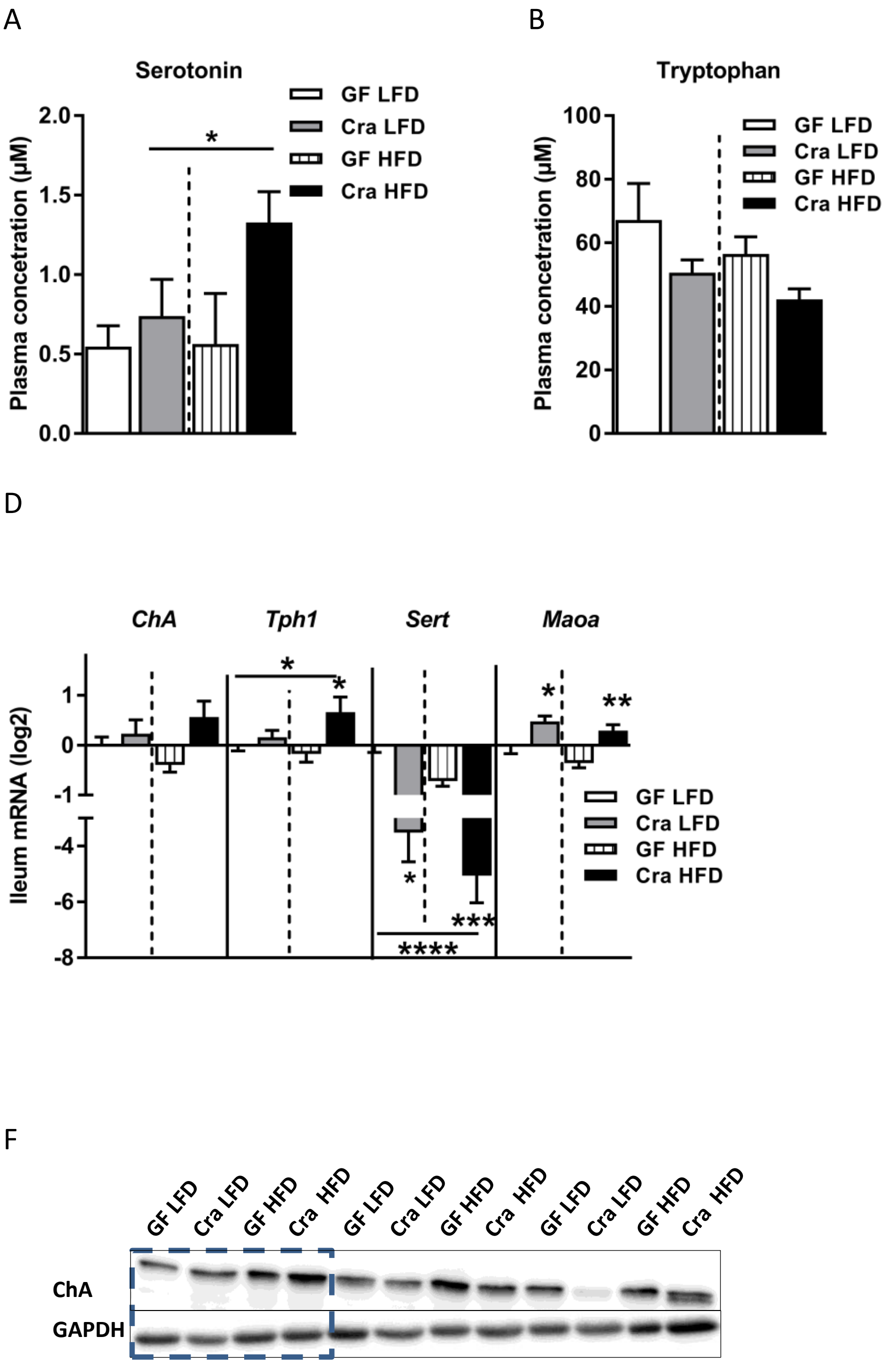

A
Serotonin
Plasma concetration (µM)
2.0
1.5
1.0
0.5
0.0
*
GF LFD
Cra LFD
GF HFD
Cra HFD
B
Tryptophan
Plasma concetration (µM)
100
80
60
40
20
0
GF LFD
Cra LFD
GF HFD
Cra HFD
C
Kynurenine/Tryptophan
Ratio of plasma conc.
10
8
6
4
2
0
***
***
GF LFD
Cra LFD
GF HFD
Cra HFD
D
ChA
Tph1
Sert
Maoa
Ileum mRNA (log2)
1
0
-1
-4
-6
-8
*
*
*
*
**
***
****
GF LFD
Cra LFD
GF HFD
Cra HFD
E
Colon
ChA protein expression
3
2
1
0
GF LFD
Cra LFD
GF HFD
Cra HFD
F
GF LFD
Cra LFD
GF HFD
Cra HFD
GF LFD
Cra LFD
GF HFD
Cra HFD
GF LFD
Cra LFD
GF HFD
Cra HFD
ChA
GAPDH

Figure S3

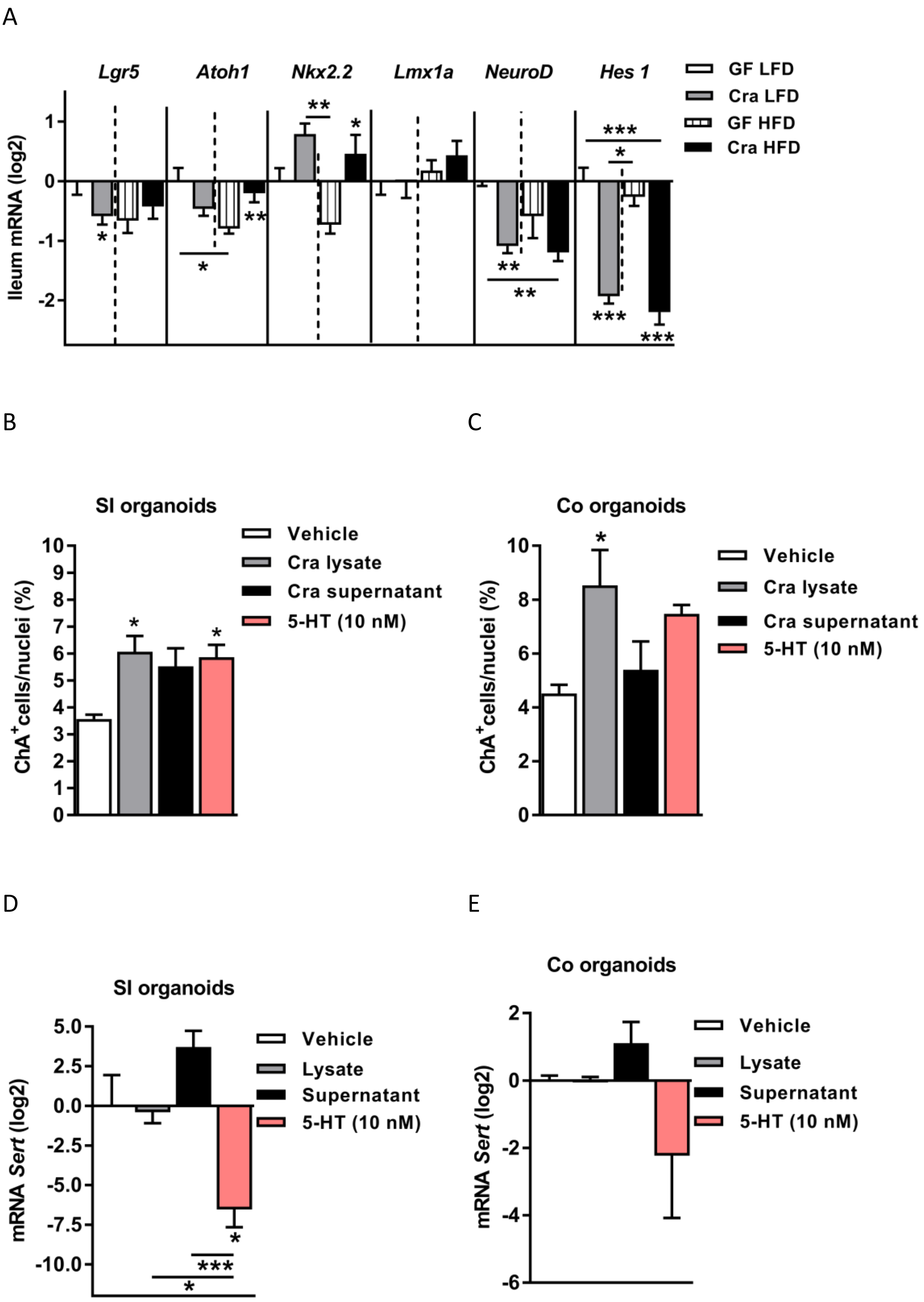
A
Lgr5
Atoh1
Nkx2.2
Lmx1a
NeuroD
Hes 1
GF LFD
Cra LFD
GF HFD
Cra HFD
Ileum mRNA (log2)
B
C
SI organoids
Vehicle
Cra lysate
Cra supernatant
5-HT (10 nM)
ChA+cells/nuclei (%)
Co organoids
D
E
mRNA Sert (log2)
Lysate
Supernatant

Figure S4

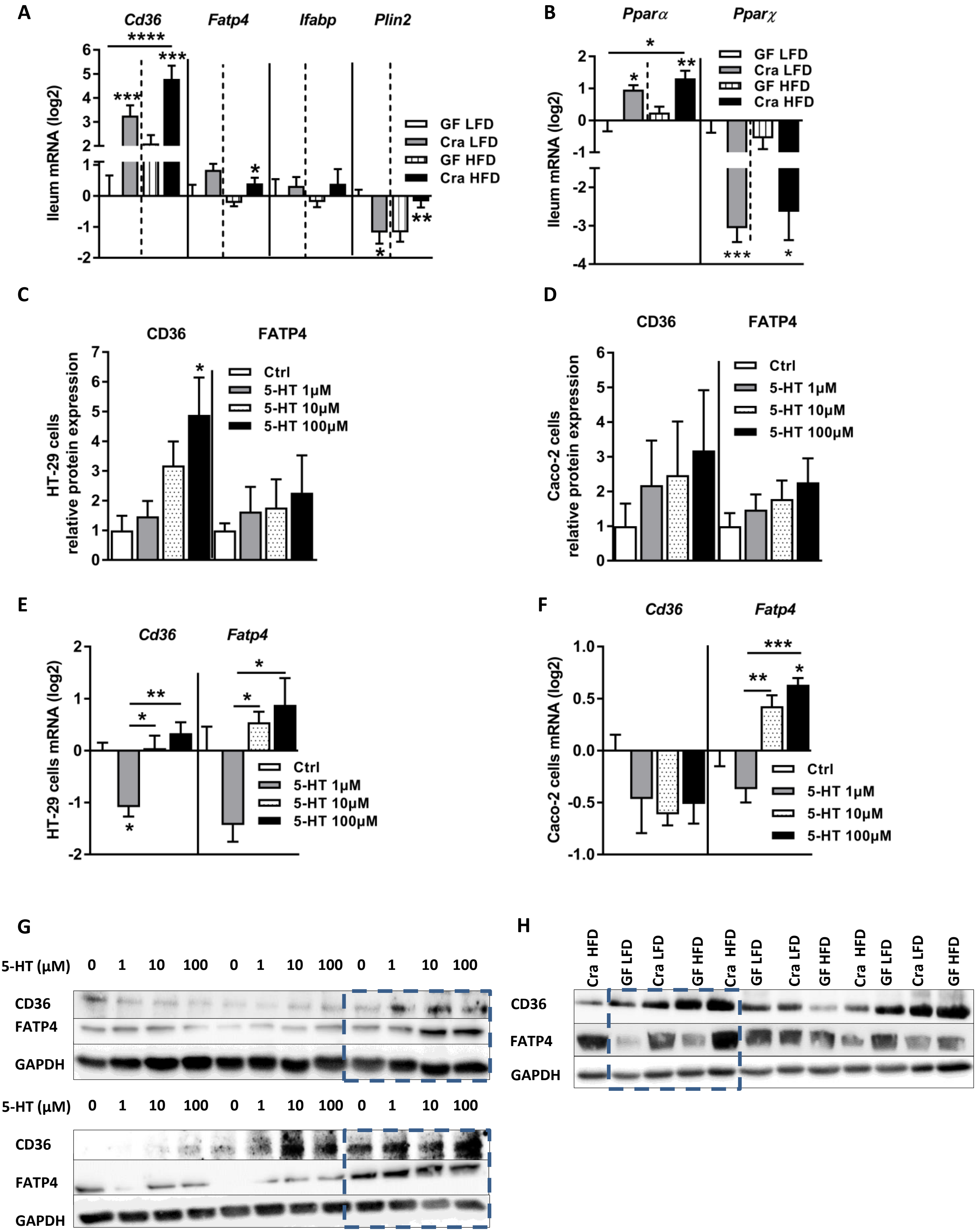

A
Cd36
Fatp4
Ifabp
Plin2
Ileum mRNA (log2)
GF LFD
Cra LFD
GF HFD
Cra HFD
B
Pparα
Pparχ
Ileum mRNA (log2)
GF LFD
Cra LFD
GF HFD
Cra HFD
C
CD36
FATP4
HT-29 cells relative protein expression
Ctrl
5-HT 1μM
5-HT 10μM
5-HT 100μM
D
CD36
FATP4
Caco-2 cells relative protein expression
Ctrl
5-HT 1μM
5-HT 10μM
5-HT 100μM
E
Cd36
Fatp4
HT-29 cells mRNA (log2)
Ctrl
5-HT 1μM
5-HT 10μM
5-HT 100μM
F
Cd36
Fatp4
Caco-2 cells mRNA (log2)
Ctrl
5-HT 1μM
5-HT 10μM
5-HT 100μM
G
5-HT (μM) 0 1 10 100 0 1 10 100 0 1 10 100
CD36
FATP4
GAPDH
5-HT (μM) 0 1 10 100 0 1 10 100 0 1 10 100
CD36
FATP4
GAPDH
H
Cra HFD
GF LFD
Cra LFD
GF HFD
Cra HFD
GF LFD
Cra LFD
GF HFD
Cra HFD
GF LFD
Cra LFD
GF HFD
CD36
FATP4
GAPDH

Figure S5

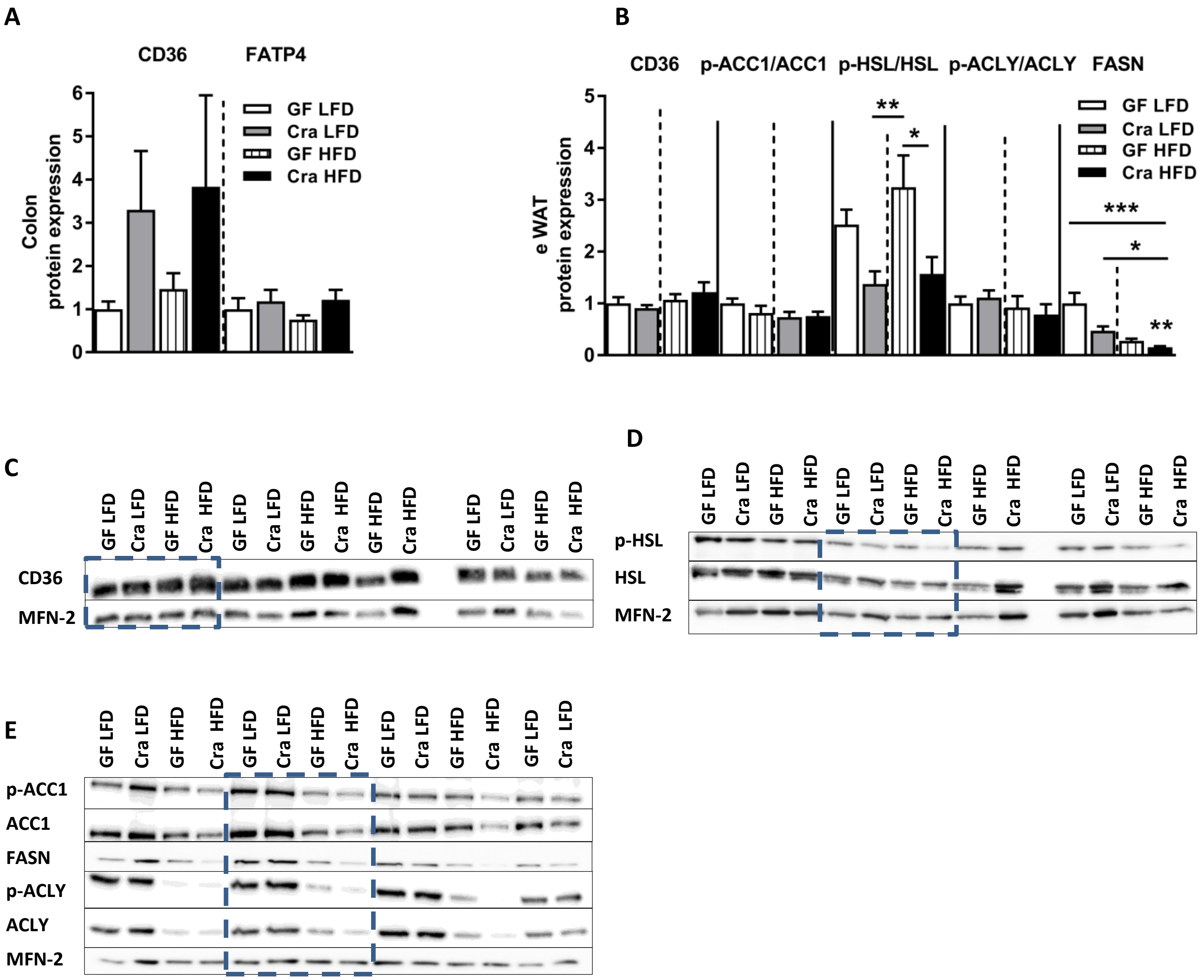
A
CD36
FATP4
Colon
protein expression
GF LFD
Cra LFD
GF HFD
Cra HFD
B
CD36
p-ACC1/ACC1
p-HSL/HSL
p-ACLY/ACLY
FASN
e WAT
protein expression
GF LFD
Cra LFD
GF HFD
Cra HFD
**
*
***
*
**
C
CD36
MFN-2
D
p-HSL
HSL
MFN-2
E
p-ACC1
ACC1
FASN
p-ACLY
ACLY
MFN-2
